# Identification of Novel Dopamine D_2_ Receptor Ligands—A Combined In Silico/In Vitro Approach

**DOI:** 10.3390/molecules27144435

**Published:** 2022-07-11

**Authors:** Lukas Zell, Constanze Lainer, Jakub Kollár, Veronika Temml, Daniela Schuster

**Affiliations:** Department of Pharmaceutical and Medicinal Chemistry, Institute of Pharmacy, Paracelsus Medical University, 5020 Salzburg, Austria; lukas.zell@pmu.ac.at (L.Z.); constanze@lainer.co.at (C.L.); jakub.kollar@pmu.ac.at (J.K.); veronika.temml@pmu.ac.at (V.T.)

**Keywords:** dopamine receptor, GPCR, in silico, pharmacophore modelling, virtual screening, in vitro, HTRF

## Abstract

Diseases of the central nervous system are an alarming global problem showing an increasing prevalence. Dopamine receptor D_2_ (D_2_R) has been shown to be involved in central nervous system diseases. While different D_2_R-targeting drugs have been approved by the FDA, they all suffer from major drawbacks due to promiscuous receptor activity leading to adverse effects. Increasing the number of potential D_2_R-targeting drug candidates bears the possibility of discovering molecules with less severe side-effect profiles. In dire need of novel D_2_R ligands for drug development, combined in silico/in vitro approaches have been shown to be efficient strategies. In this study, in silico pharmacophore models were generated utilizing both ligand- and structure-based approaches. Subsequently, different databases were screened for novel D_2_R ligands. Selected virtual hits were investigated in vitro, quantifying their binding affinity towards D_2_R. This workflow successfully identified six novel D_2_R ligands exerting micro- to nanomolar (most active compound K_I_ = 4.1 nM) activities. Thus, the four pharmacophore models showed prospective true-positive hit rates in between 4.5% and 12%. The developed workflow and identified ligands could aid in developing novel drug candidates for D_2_R-associated pathologies.

## 1. Introduction

Diseases of the central nervous system in general show a trend towards an increasing prevalence. Neurodegenerative diseases represent an especially alarming global problem, which is directly linked to the increased life expectancy. The family of G protein-coupled receptors (GPCRs) represents the most important drug targets for currently approved drugs and is still a rich target resource in current drug research [1]. Various subgroups of GPCRs are known to be centrally involved in central nervous system diseases [2]. One of the most prominent members of the GPCR family is the dopamine receptor (DR) family, which is involved in Parkinson’s disease, schizophrenia, bipolar disorders, and other pathologies. Throughout the last decades, 65 drugs targeting the DR family have been approved by the US Food and Drug Administration (FDA) [3]. DR-targeting drugs affect their targets with a variety of different modes of action, for example, DR-antagonistic antipsychotics, DR partial agonists used in bipolar disorders and addiction, and antiparkinsonian DR agonists [4,5,6,7].

While all DR subtypes are highly interesting, DR D_2_ (D_2_R) especially has been shown to be involved in all of the conditions listed above. Out of the 65 FDA-approved drugs, 60 also target D_2_R [3]. Current therapies of PD, for example, involve D_2_R ligands levodopa or apomorphine [8,9]. Amoxapine, aripiprazole, and haloperidol are used in the treatment of depression, psychosis, or schizophrenia [10]. While these drugs are successful in treating the respective conditions, they all suffer from major drawbacks. Dopamine receptor agonists such as levodopa initially improve motor symptoms in Parkinson’s disease patients, reducing so-called off periods. However, levodopa therapy efficacy decreases with time. Moreover, it is problematic in chronic settings due to treatment-induced dyskinesia and other levodopa-induced motor symptoms such as postural abnormalities and speech impairments [11]. Moreover, levodopa treatment involves non-motor side effects such as nausea, depression, insomnia, and gambling addiction [12]. Apomorphine is generally well-tolerated in terms of non-motor symptoms. However, its safety and efficacy suffers from low bioavailability due to intense first-pass metabolism and adverse skin reactions depending on drug administration [8]. These side effects during treatment of Parkinson´s disease with levodopa or apomorphine can lead to a reduction in the quality of life and, subsequently, to discontinuation of the therapy. The use of D_2_R antagonists as antipsychotics in several DR-related psychiatric disorders is a standard therapeutic approach. However, these antipsychotics are also tightly associated with adverse side effects such as extrapyramidal syndrome and neuroleptic malignant syndrome [13,14]. The high prevalence of side effects in the treatments targeting D_2_R is also due to the promiscuity of the used therapeutics [15]. None of the 60 drugs approved by the FDA are truly selective for D_2_R but are also associated with other members of the DR family [3]. Moreover, D_2_R-targeting agents also act on closely related receptors from other GPCR families such as different subtypes of the adenosine or serotonin receptor families [16]. The last successful approval of a drug targeting D_2_R was levodopa in 2015. Several clinical trials have been discontinued due to insufficient efficacy. Four trials investigating dopamine receptor agonists in Parkinson´s disease (PF-06412562, PF-06669571, PF-06649751, and CJH1) are currently ongoing [17]. This illustrates the high demand for novel D_2_R-targeting agents.

Drug research regarding GPCR-targeting agents has been driven by the so-called “golden age for GPCR structural biology” in cheminformatics [18]. High-resolution 3D structures with bound ligands provide the means for rational structure-based (SB) drug design. As of today, there are five 3D structures available in the Protein Data Bank (PDB). Three X-ray structures are bound to an antagonist (6cm4 [19] bound to risperidone, 7dfp [20] bound to spiperone, and 6luq [21] bound to haloperidol). Two cryo-EM structures are bound to agonists (6vms [22] and 7jvr [23], both in complex with bromocriptine). They further contribute to understanding the receptor–ligand interaction and the mode of action for D_2_R. The most recent entry 7jvr has not been utilized in SB virtual screening yet. While SB approaches are highly valuable, they are limited by the scarcity of available crystal structures considering GPCRs in general and specifically D_2_R. Thus, ligand-based (LB) virtual screening approaches present an important complementary approach. They exploit the availability of an ever-growing number of drug-like small molecules offering a spectrum of chemical scaffolds with known biological and pharmacological profiles [24]. This was shown in different studies investigating D_2_R agonists. Therefore, the combination of SB and LB virtual screening approaches is recommended to cover a broad chemical space of active compounds in screening campaigns [25,26,27,28,29,30,31,32,33,34,35,36,37,38].

**Figure 1:**
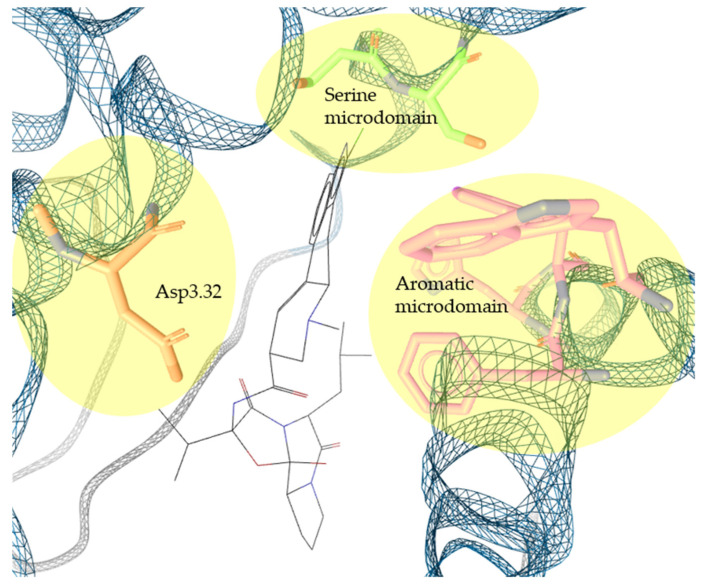
Overview of the highly conserved D_2_R orthosteric binding pocket. Asp3.32, the serine microdomain, and the aromatic microdomain responsible for ligand binding, orientation, and receptor activation are highlighted. Picture generated in LigandScout (based on cryo-EM structure 7jvr [23]) showing bromocriptine in the orthosteric binding pocket.

Virtual screening approaches investigating D_2_R also greatly benefit from the pioneering work of Floresca and Schentz in describing the orthosteric binding pocket (shown in Figure 1) of the receptor [39]. To enable direct comparisons of different receptor-binding pockets, they relied on the Ballesteros–Weinstein numbering [40]. Applying this approach (e.g., X.50), amino acid positions are annotated based on the amino acid’s position within one of the seven (X = 1 to 7) transmembrane segments (TMs) typically present in GPCRs. To indicate the specific position of the amino acid of interest in the respective TM, X.50 annotates the amino acid in reference to the most conserved one. Thus, one of the most crucial amino acids in ligand binding in the DR family is annotated as Asp3.32. Asp3.32, a negatively charged aspartate within the DR orthosteric binding pocket, was found to be highly conserved not only within DR members, but essentially in all aminergic GPCRs [32,41,42]. Asp3.32 represents the negatively ionizable partner of the key PI of the ligand. To successfully form this crucial ionic bond, the DR ligand needs to contain a protonable nitrogen. This is true not only for the endogenous ligand dopamine; a study by Bueschbell et al. showed that the Asp3.32 interaction is present in essentially all D_2_R ligands [43]. Interestingly, the ionic bond is present independently of the ligand’s mode of action, i.e., agonist (such as apomorphine) or antagonist (such as haloperidol). Investigating D_2_R, the essential ionic bond corresponds with Asp114. Another important structural element that influences ligand affinity and orientation is the serine microdomain located in TM 5 [39,42,44,45,46]. Ser5.42, 5.43, and 5.46 (highly conserved in the DR family) corresponding with Ser193, 194, and 197 in the D_2_R structure form key hydrogen bonds (HBs). In dopamine binding, these HBs are formed with a catechol functional group. In other known D_2_R ligands, HBs are present as well. However, the interaction with the serine microdomain is less dominant. This was elucidated by Bueschbell et al. showing larger distances between the serine microdomain and the ligand in the absence of catechol functionality [43]. Thus, binding affinity appears to be affected by the formation of HBs, but not necessarily by distinct amino acid interactions. Asp114 and formed HBs are responsible for ligand binding but do not determine the ligand’s mode of action. However, different studies defined another microdomain, the aromatic microdomain, involved in the so-called “rotamer toggle switch”, leading to receptor activation upon ligand binding [39,41,47]. This aromatic region includes amino acids like Trp6.48, Phe6.51, Phe6.52, and His/Asn6.55 corresponding to Trp386, Phe389 and 390, and His393 in D_2_R. The involvement of the aromatic microdomain in receptor activation was also confirmed by Bueschbell et al. where known D_2_R agonists such as dopamine, apomorphine, bromocriptine, and 7-OH-DPAT showed interactions with all crucial activating amino acids in the aromatic microdomain [43]. In contrast, antagonists like haloperidol, risperidone, or aripiprazole lacked some of the amino acid interactions. Additionally, Floresca and Schnetz also reported that amino acid residues Trp386 and His393 act as stabilizers of agonists via π–π stacking [39].

In silico approaches need to be combined with in vitro testing for computational model validation and hit verification. Binding affinities of potential D_2_R ligands can be investigated utilizing different in vitro methods, e.g., homogenous time-resolved fluorescence (HTRF) assays [48,49]. This assay type is easily standardizable and commercially available for DR family members.

**Figure 2:**
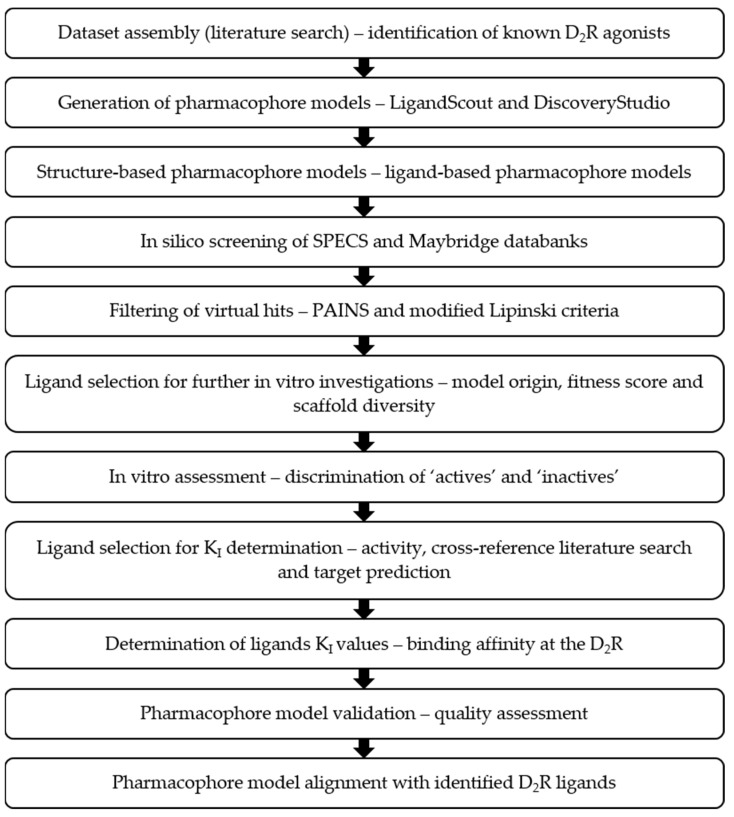
Combined in silico/in vitro approach to identify novel D_2_R ligands.

The aim of this study was to develop and validate high-quality in silico pharmacophore models for D_2_R ligands and validate them in vitro. Providing novel D_2_R ligands using our combined workflow would contribute to the discovery of novel pharmacological tools and starting points for further drug development. In Figure 2, a rational approach in identifying novel D_2_R ligands is shown.

## 2. Results

### 2.1. Dataset Assembly

**Figure 3:**
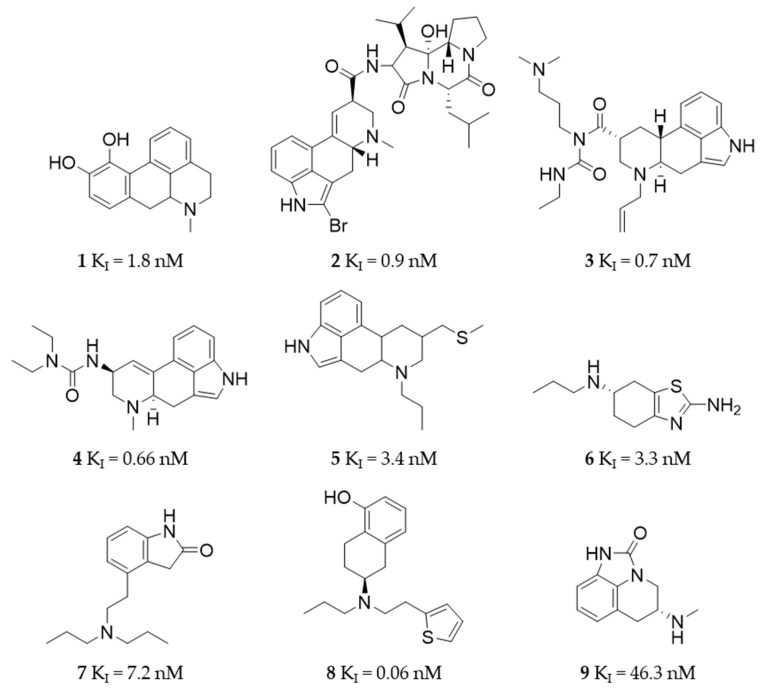
Overview of all clinically used D_2_R agonists. All compounds were included in the active dataset used during the generation and validation of the developed pharmacophore models. The compounds were retrieved from the ChEMBL database including their in vitro K_I_ values (nM).

To generate a high-quality dataset for the generation of the pharmacophore models, highly active D_2_R ligands included in the ChEMBL database were compiled (Figure 3 and Table S1) [50,51,52,53,54,55,56,57,58,59,60,61,62,63,64,65,66,67,68,69,70]. The final set included 68 active compounds. The clinically used D_2_R agonists (**1**–**9**) are shown in Figure 3.

The dataset of inactives (**SC60**–**SC127**) was also derived from the ChEMBL and comprised 68 compounds with a K_I_ higher than 50 µM. The inactive dataset is shown in Table S2 [71,72,73,74,75,76,77,78,79,80,81,82,83,84,85,86,87,88,89,90,91,92]. The decoy dataset including 3752 compounds was generated utilizing the DUD-E webtool (http://dude.docking.org/ [93], accessed on 15 March 2021) and based on the structures of the active dataset.

### 2.2. Pharmacophore Models—LB and SB Approaches in LigandScout and DiscoveryStudio

Pharmacophore models were generated using two different software packages (LigandScout (LS) [94] and DiscoveryStudio (DS) [95]) employing SB and LB approaches. The different software packages utilize distinct screening algorithms. While the different algorithms result in different screening results, it has been shown that they are complementary, eventually increasing the coverage of the chemical space of D_2_R ligands [96]. All pharmacophore models were initially automatically generated by the respective software. Subsequently, their individual performances were manually optimized to improve their capability of correctly distinguishing between active and inactive compounds [97]. In this step, all pharmacophore features (positively ionizable interaction (PI), hydrogen bond acceptor and donor (HBA and HBD), hydrophobic contact (HC), aromatic interaction (AI), exclusion volumes (XVOLs)) were individually optimized. Their tolerances were adapted to maximize the number of found active compounds while minimizing the number of inactive compounds and decoys, respectively. This improvement in model performance was primarily achieved by enhancing model restrictiveness, i.e., by decreasing the tolerance sphere of the PI, HBA, HBD, HC, or AI features, increasing the XVOLs’ size, completely removing specific XVOLs or a selected feature. XVOLs were also added to all the models to increase model specificity. If this additional XVOL eliminated some inactive compounds or decoys but did not result in a decrease in sensitivity, it was included. Eventually, enrichment factor (EF) and yield of actives (YoA) were used as optimization parameters during the evaluation process.

**Figure 4:**
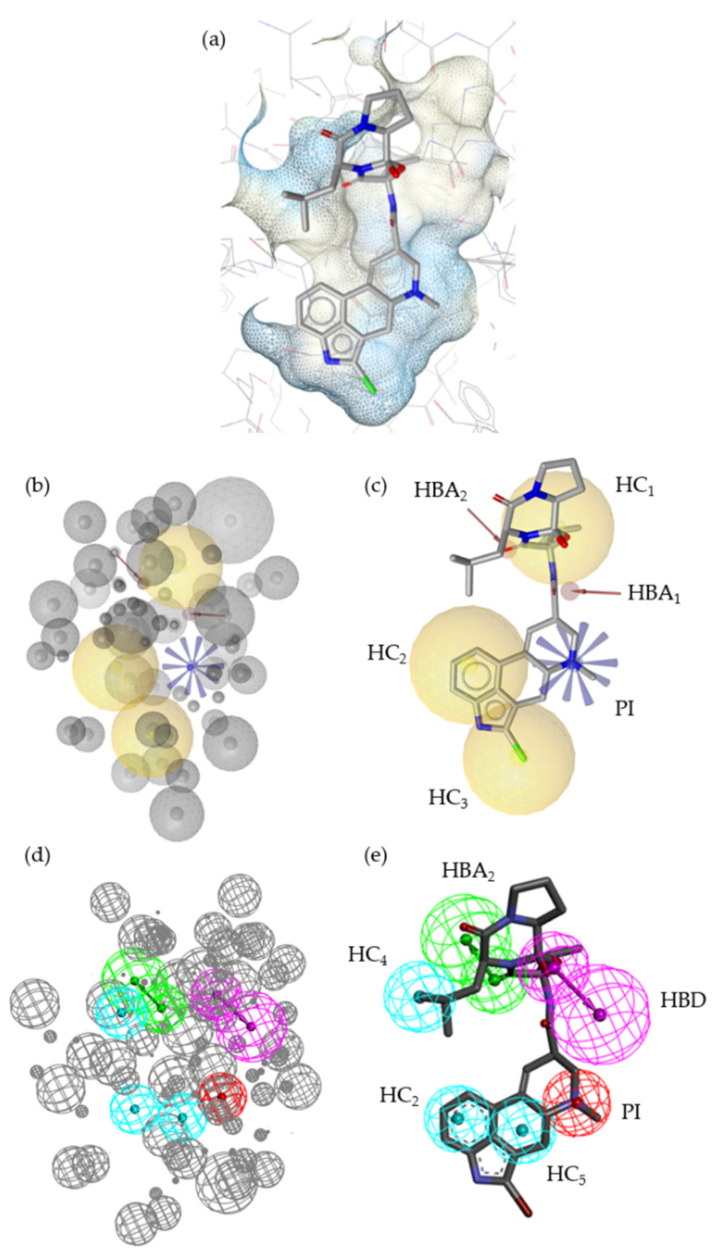
SB pharmacophore models M1 and M2 generated using LS and DS, respectively. M1 and M2 are based on the cryo-EM structure of D_2_R in complex with agonist **2** (PDB ID 7jvr [23]). (**a**) Agonist **2** in the ligand-binding pocket of D_2_R. (**b**) M1 including 60 exclusion volumes (XVOLs). (**c**) M1 aligned with **2**. (**d**) M2 including 113 XVOLs. (**e**) M2 aligned with **2**. XVOLs (grey). Hydrophobic contacts (HCs; yellow and cyan spheres). Hydrogen bond acceptor (HBA; red arrows, green spheres). Hydrogen bond donor (HBD; purple sphere). Positively ionizable interaction (PI; blue star-like shape, red sphere). All features represent characteristics of the respective structural features of the investigated molecules.

The SB models shown in Figure 4 (LS SB, M1; DS SB, M2) were both based on the recently published cryo-EM structure of D_2_R in complex with agonist bromocriptine (**2**; PDB ID 7jvr [23], display of **2** in the D_2_R-binding pocket (Figure 4a)). An LB model was generated in each software. The resulting models were then optimized with the datasets of actives, inactives, and decoys within their software environment. The full optimization workflow is shown in Tables S3 and S4.

While both automatically generated models were nearly identical (Figure S1), they differed significantly after the optimization process. The SB models shared similar features (Figure 4b–e). Both SB models contained one PI feature, three HC features, and two HB features. However, the distribution of the HC features differed between the models. HC_1_ was located in the tricyclic ring system in M1. In M2, HC_1_ was not present. HC_2_ and HC_3_ were located in the tetracyclic part of **2** in M1. HC_4_ was located at the isobutyl moiety of **2** in M2. Moreover, M1 included two HBA features (HBA_1_ and HBA_2_) while M2 included one acceptor (HBA_2_) and one HBD feature. The coordinates of HBA_2_ were identical in both SB models. HBA_1_ and HBD differed considering coordinates and feature type. Additionally, both models included distinct XVOLs, which were independently and manually optimized within LS and DS.

The LB models were based on compounds **1**, **SC52**, and **SC59** (Figure S2). Those compounds were selected since they were amongst the most active ones (K_I_ < 2 nM), structurally diverse, and mostly not identified by M1 and M2. The LB models were generated in each software and optimized as described above (for the optimization details see Tables S5 and S6).

**Figure 5:**
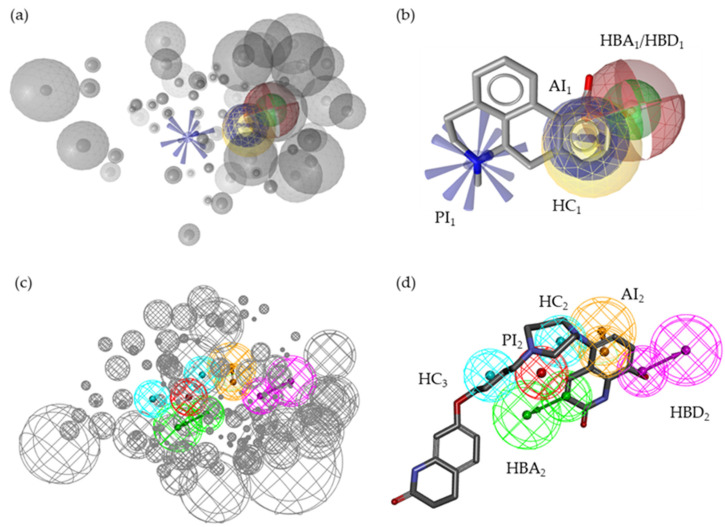
Ligand-based pharmacophore models M3 and M4 generated using LS and DS, respectively. Both models were based on **1**, **SC52**, and **SC59**. (**a**) Display of M3 including 61 exclusion volumes (XVOLs). (**b**) M3 aligned with **1**. (**c**) Display of M4 including 122 XVOLs. (**d**) M4 aligned with **SC59**. XVOLs (grey). Hydrophobic contacts (HCs; yellow and cyan spheres). Hydrogen bond acceptor (HBA; red and green spheres). Hydrogen bond donor (HBD; green and purple sphere). Positively ionizable interaction (PI; blue star-like shape, red sphere). Aromatic interaction (AI; blue circle, orange sphere). All features represent characteristics of the respective structural features of the investigated molecules.

Although both LB models were initially based on the same compounds, they differed distinctly from the beginning (the original models are shown in Figure S3). After optimization (LS LB, M3, and DS LB, M4; Figure 5), both models contained one PI feature, two HB features, one AI feature, and at least one HC feature. While the PI features (PI_1_ and PI_2_) and the AI features (A_1_ and A_2_) were comparable to each other, other features were distinctly different between the models. Although both models featured two HBs, they were superimposed in M3, while HBA_2_ and HBD_2_ were clearly located at different coordinates. Furthermore, HC_1_ and AI_1_ in M3 were superimposed. In contrast, AI_2_ and HC_2_ were located at different coordinates in M4. Moreover, M4 contained an additional HC feature (HC_3_). Comparability of the pharmacophore features models was assessed based on **SC59** which was included in the hitlist of both optimized LB models (shown in Figure S4). Additionally, both models included distinct XVOLs, which were independently and manually optimized within LS and DS.

In Table 1, the evaluation of the four pharmacophore models is summarized. Each model found one inactive compound. All of the models found a small number of decoys. Moreover, none of the pharmacophore models found all of the 68 active compounds. However, a sufficient yield of actives combined with small false-positive hit rates resulted in high model accuracy of all the four pharmacophore models in between 0.98 and 0.99.

While all the pharmacophore models were defined by high accuracy, they distinctly differed in EF and YoA. M3 clearly showed the highest EF as well as YoA. Moreover, all the models were characterized by high specificity and sensitivity ranging from 0.13 (M1) to 0.44 (M4). In general, the LB models M3 and M4 performed better considering all quantitative parameters. All the pharmacophore models combined identified 51 unique actives, covering 75% of the actives dataset. At the same time, only two molecules defined as inactive and 108 decoys were found by the models. While specificity, accuracy, EF, and YoA were well within the range of the separate models, the performance of the combined models was characterized by an increased sensitivity of 0.75.

### 2.3. Screening of the SPECS and Maybridge Databases

A total of 260,743 compounds (208,968 originating from SPECS, 51,775 originating from the Maybridge databank) were screened. All virtual hits were filtered using a “rule of five”-based physicochemical descriptor filter (Ro5; settings shown in Section 4.4) as well as the PAINS (PAINS1, 2, and 3) criteria [98,99]. Filtering resulted in 2240 hits from the different pharmacophore models. Table 2 shows the number of hits that were found by each model in the different databases. The Ro5 and PAINS filters were applied using a combined script in PipelinePilot [95].

**Figure 6:**
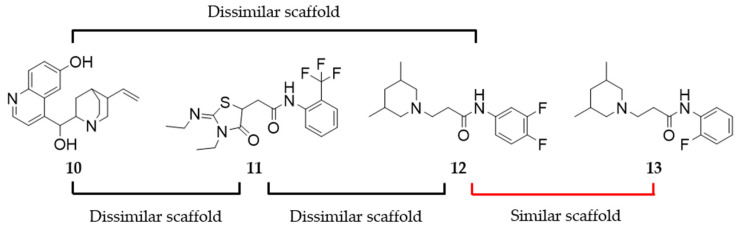
Exemplary assessment of scaffold similarity of consensus hits (n = 3).

The compounds were ranked according to the number of models they were found by. Furthermore, virtual hits in all of the groups were sorted according to the fit score of the respective model by which they were found. In consensus hits, the model with the highest fit score was selected as a reference. Triple consensus hits, the virtual hits found by M2, M4, and M1/M3, had the highest priority. These virtual hits were followed by double consensus hits including hits from M2/M4 and M1/M3 and consensus hits from M2 and M4. No virtual hit was found by both of the LS models (M1 and M3). Consequently, category M1/M3 was introduced in the consensus hit categories, indicating the hits identified by M1 or M3. A table detailing which compounds were found by which model is shown in the Supplementary Information (Table S7). Scaffold diversity within all the groups was investigated. Similar scaffolds within one group were discarded following the principle (comparing **10**–**13**) shown in Figure 6.

Scaffold similarity was not considered between the different hit groups. Compound selection of the single-model virtual hits was conducted based on the same criteria already described considering the consensus hits. In addition to most consensus hits, 16 distinct virtual hits were selected from each model (M1–M4), allowing for balanced model validation. Scaffold similarity was additionally analyzed (Table S8 and Figure S5) applying Tanimoto scores (TS) [100], which confirmed the structural diversity of the compounds selected for biological testing. Compound **14** was prioritized for in vitro investigation because of its high similarity to the previously marketed drug octoclothepine, indicating a high probability to be a true D_2_R ligand.

### 2.4. In Vitro Compound Screening

Activity of the selected virtual hits was investigated via competitive binding (in comparison to a fluorescence-labeled ligand) of the respective compounds at D_2_R utilizing an HTRF assay. From the selected virtual hits, several compounds showed a normalized decrease in fluorescence equal or higher than 2 (Table 3). A complete summary of the in vitro binding assessment of the 90 selected virtual hits is shown in Figure S6 and Table S9, including the compound structures. All the normalized decrease in fluorescence values of the known D_2_R ligands (control compounds) as well as the compounds considered active are summarized in Table 3.

**Figure 7:**
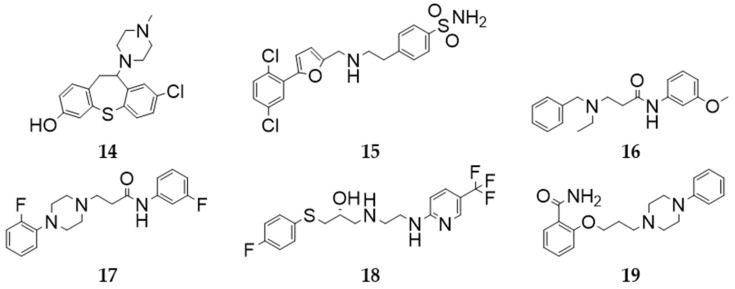
Two-dimensional structures of the compounds selected for in vitro K_I_ determination.

Only compound **16** showed clear discrepancies in between the replicates of the in vitro screening. Subsequently, the respective compounds were investigated considering structural novelty and lack of information about the D_2_R interaction to select the most interesting candidates for K_I_ determination. Based on this analysis, compounds **14**, **15**, **16**, **17**, **18**, and **19** were selected for K_I_ determination on D_2_R (the selected compounds and the potential candidates are shown in Figure 7 and Figure S10, respectively).

### 2.5. K_I_ Determination—D_2_R Binding Affinity of the Selected Compounds

**Figure 8:**
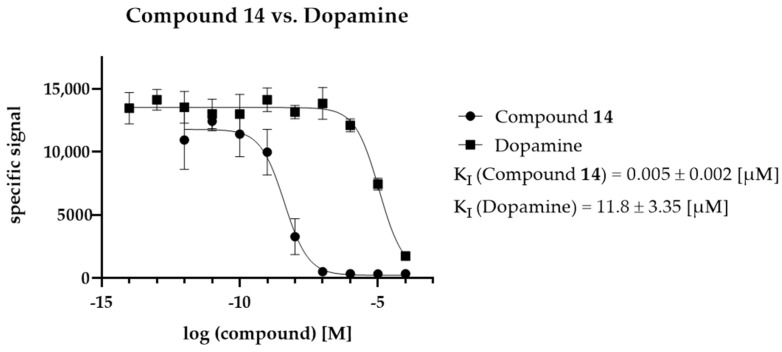
Comparison of the K_I_ values of dopamine (endogenous ligand) and compound **14** (highest activity of the identified ligands). K_I_ values were determined with n = 6.

The selected compounds were investigated in vitro to determine their binding affinities (K_I_ values) at D_2_R. In Figure 8, compound **14**, exerting the highest activity of all the investigated compounds, is shown in comparison to dopamine as the endogenous D_2_R ligand. The remaining compounds in comparison to dopamine are shown in Figure S7.

The activity of compound **16** was similar to that of dopamine. All the other compounds showed activities in the micromolar (compounds **15**, **17**, and **18**), submicromolar (compound **19**), and even nanomolar (compound **14**) range. The respective K_I_ values including their confidence intervals are shown in Table 4.

### 2.6. In Silico Model Evaluation

The six investigated compounds (out of 90) showed at least a similar or even higher affinity towards D_2_R compared to the endogenous ligand dopamine. To analyze the quality of the in silico pharmacophore models, the hit rate (%) of each model was calculated (referring to Table S7). All the compounds but one were consensus hits from different combinations of the pharmacophore models. Only compound **18** was identified by a single model (M2). Moreover, all the four pharmacophore models identified at least one active D_2_R ligand. The quantitative predictive power of the individual pharmacophore models is shown in Table 5.

While all of the generated pharmacophore models identified at least one novel D_2_R ligand, differences could be observed between the models regarding predictive power. While M1 successfully identified one ligand, the ligand-based models M3 and M4 identified three active D_2_R ligands. M2 identified four novel D_2_R ligands out of the selected virtual hits. In total, the hit rate of the pharmacophore models ranged between 4.5% and 12%. According to a similarity analysis, the novel active D_2_R ligands showed low similarity with the literature dataset (Table S11), with a maximum TC of 0.26 (compound **19** and **SC30**). Additionally, a complete Tanimoto matrix comparing all the ligands and training compounds is shown in Figure S8.

### 2.7. Alignment of the Pharmacophore Models with Novel D_2_R Ligands

**Figure 9:**
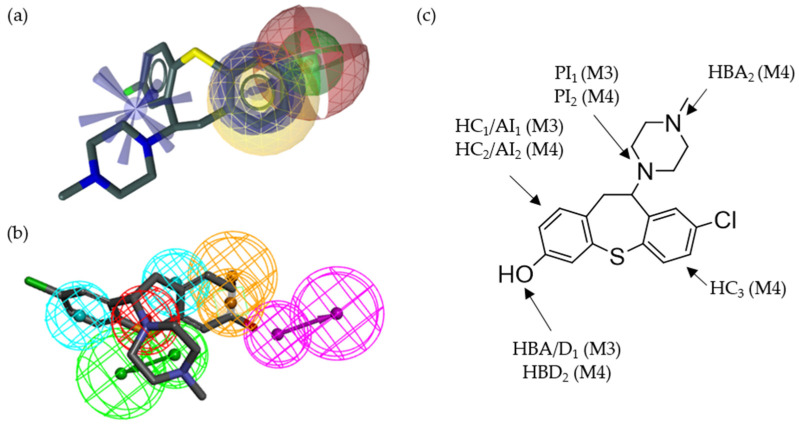
Display of the novel D_2_R ligand, compound **14**, aligned with M3 and M4, identifying the compound during the screening process. (**a**) Three-dimensional structure of compound **14** including the pharmacophore features of M3. (**b**) Three-dimensional structure of compound **14** including the pharmacophore features of M4. (**c**) Two-dimensional structure of compound **14** highlighting the structural features recognized by the pharmacophore models.

Structural features of the identified D_2_R ligands were mapped to the respective pharmacophore models. Exemplarily, the mapping of compound **14** is shown in more detail in Figure 9. Both models placed the AI feature on the phenolic ring of dibenzothiepin. HC_1_ and HC_2_ are located at similar coordinates as the AIs in both models. HC_3_, only present in M4, was located on the chloro-substituted ring of dibenzothiepin. The HB feature located on the hydroxy group was present in both models. However, it was defined as an HBD feature in M4, while M3 allowed both acceptor and donor features. M4 featured an additional HB feature (HBA_2_) located on the methylated tertiary amine of piperazine. Most importantly, both models placed the PI on the connecting tertiary amine of piperazine. The mapping and alignment of compounds **15**, **16**, **17**, **18**, and **19** is available in the Supporting Information (Figures S9–S13).

### 2.8. Assessing the Scaffold Novelty of the Identified Ligands Compared to Known D_2_R Ligands in ChEMBL

Comparing the identified D_2_R ligands to their most similar ChEMBL entries, respectively, the scaffolds of compounds **14**, **17**, and **19** are already known in the literature. Compound **14** is characterized by the highest TS (0.58) since it only features an additional hydroxy group in comparison to the most similar ChEMBL entry. In compound **15**, the amine–furane–benzyl substructure is known. However, the sulfonamide functionality appears to be novel. The scaffold of compound **19** is already known in the literature. However, the ether functionality appears to be novel. Additionally, amide functional groups present in compounds **16**, **17**, and **19** are already known. An overview of the similarity analysis is shown in Figure S8 and Table S12.

## 3. Discussion

The theoretical evaluation of the pharmacophore models based on EF and YoA (shown in Table 1) revealed M3 as the top-ranking model. While M4 and M2 were ranked second and third (although very similar to each other regarding their performance), M1 clearly performed the worst. This evaluation of the model performances was confirmed during experimental validation (summarized in Table 5). M3 was top-ranked, with a hit rate of 12%, while M1 showed a hit rate of 4.5%. M2 and M3 were the most comprehensive models as their combined hitlist included all the active compounds (see Table S7). M4 performed equally to M3 considering the number of active compounds found during prospective screening. M1 alone identified only one active compound. There was no difference in performance regardless of whether the model was SB or LB. Most active hits (5/6) were consensus hits, suggesting that a combination of the various models increases true-positive hit rates. This synergistic effect of LB and SB screening is also in agreement with the current literature [101]. The same observation was made when investigating the synergistic combination of the different modelling environments LS and DS. Again, all the compounds except compound **18** were successfully identified due to a combination of the models generated in both programs.

Ligand novelty was investigated utilizing structure similarity searches in SciFinder searching for both identical or similar compounds and their interaction with the DR family in general and specifically D_2_R. While the search did not yield any exact structural matches, some scaffolds have already been associated with DR activity in the literature.

A similar structure to compound **14** (SKI-417616) was investigated as a dopamine D_4_ receptor (D_4_R) antagonist, as well as its role in the inhibition of dengue virus replication [102]. However, the study did not investigate SKI-417616 in vitro but based its D_4_R antagonistic potency on earlier studies investigating pyrrolobenzothiazepine derivatives [103]. However, SKI-417616 has not been investigated considering D_2_R. Naporra et al. identified novel dibenzoxazepine and –oxepine derivatives binding to both the D_2_R_long_ and D_2_R_short_ variants. However, none of the analyzed compounds included a thioether functional group. Therefore, compound **14** represented a different scaffold. A 3D-QSAR (quantitative structure–activity relationship) study presented a retrospective study based on known D_2_R and D_4_R antagonists. However, the investigated compounds, again, did not include the thioether group present in compound **14**. The role of similar compounds acting as insecticides through D_1_R antagonism was investigated by Meyer et al. This study focused only on non-human DRs from the mosquito *Aedes aegypti* [104,105,106]. In agreement with the SciFinder literature search, the most similar compound found (shown in Table S12) was octoclothepine (**SC209**, brand names Clotepin and Clopiben), a highly potent D_2_R antagonist that was also clinically used for a short time in 1971 for the treatment of schizophrenic psychosis [107].

Compound **15** has not been reported as a DR ligand according to the SciFinder results. **SC210**, the most similar compound found in ChEMBL (4-(5-(((2-(5-fluoro-1*H*-pyrrolo[2,3-b]pyridin-3-yl)ethyl)amino)methyl)furan-2-yl)phenol, TS of only 0.21) was an antagonist for D_2_R identified in a virtual screening campaign investigating serotonin receptors [108].

Referring to the similarity search in ChEMBL utilizing the Tanimoto matrix shown in Table S12, both compounds **16** and **17** showed the highest structural similarity with the same compound, **SC211**, a piperazine benzamide derivative (3-(4-(4-chlorophenyl)piperazin-1-yl)-N-(3-methoxyphenyl)propanamide, CHEMBL329228). Interestingly, the identified compound has been shown to possess high affinity and selectivity towards D_4_R [109]. Considering compound **18**, similarity searches in ChEMBL resulted in finding the most similar structure to be 1-(3-((4-fluorophenyl)thio)propyl)-4-(4-(trifluoromethyl)phenyl)piperazine (**SC212**, ChEMBL1940410), a haloperidol derivative. This compound has been associated with atypical antipsychotic activity [110]. Interestingly, compound **18** yielded the lowest TS of all the novel D_2_R ligands, with only 0.19. While it is clearly active at D_2_R, it also represents the most novel scaffold, closely followed by compound **15**.

Considering compound **19**, the ligand also has not been discussed in the literature yet. However, similar structures have been investigated, analyzing their role as D_3_R-selective compounds and D_2_R antagonists according to a SciFinder search [111]. Additionally, ChEMBL cross-referencing resulted in a highly similar compound **SC213** (1-(3-phenoxypropyl)-4-phenylpiperazine, ChEMBL1940414) missing the amide functional group, while the remaining structure was identical. **SC213** originates from the same source as **SC211** where both entries are shown to be based on haloperidol, a potent D_2_R antagonist [110]. Considering the novel D_2_R ligands which were identified, scaffold similarity searches and literature cross-referencing indicated a potential role not just as D_2_R ligands, but also as other DR family members. Moreover, the referenced studies highlight the antagonistic mode of action for most of the compounds discussed above.

The PI is a key feature in all the active compounds represented by the basic nitrogen atom. This is highlighted in the alignments shown in Figure 9 and Figure S9–S13. Two of the novel compounds, **14** and **18** (shown in Figure 9 and Figure S12), include a hydroxy functional group, potentially interacting with the serine microdomain. All of the other novel compounds contain at least two or even three structural elements enabling other HB interactions, e.g., amide or sulfonamide functional groups. The presence of such alterative binding modes rationalizes the necessity of complementary pharmacophore models for comprehensive virtual screening campaigns.

The developed combined workflow focused on the identification of ligands binding to D_2_R. This can be seen as the first step in a drug discovery campaign. From a therapeutic point of view, D_2_R agonists and antagonists are interesting agents for the treatment of different diseases as described above. Therefore, the developed workflow combined with HTRF assays can be a valuable starting point in future studies. Follow-up projects specifically investigating a(nta)gonistic behavior as well as receptor promiscuity issues addressed earlier will be conducted based on the identified D_2_R ligands. Those projects will also involve combinational approaches of in silico and in vitro tools. The already established HTRF principle presented in this manuscript will also enable in vitro selectivity quantification for D_2_R-related GPCRs; e.g., against D_1_R, D_3_R, serotonin, and adenosine receptors. Quantifying a ligand’s mode of action in vitro will require functional assays, potentially involving downstream signal quantification; e.g., Ca^2+^ flux or cAMP levels in cell-based setups.

Summarizing, we could confirm our original hypothesis showing that a high-quality combined in silico/in vitro approach enables efficient identification of novel D_2_R ligands. Questions like selectivity and mode of action of ligands still need to be addressed. Furthermore, they could drive the development of pharmacological tools, improving drug design processes in D_2_R-associated pathological conditions.

## 4. Materials and Methods

The general workflow used to follow a rational experimental design is shown in Figure 2.

### 4.1. Materials

Dopamine hydrochloride, (*S*)-(−)-sulpiride, dimethyl sulfoxide (DMSO), and droperidol were all acquired from Sigma-Aldrich. Haloperidol and piribedil were both acquired from TCI Chemicals. Bromocriptine mesylate was acquired from VWR. Apomorphine hydrochloride was kindly provided by EVERPharma AT GmbH within the context of a different project. Tested compounds (shown in Table S9) were acquired either from SPECS (https://www.specs.net/, accessed in April 2021) or Maybridge (https://www.thermofisher.com/at/en/home/industrial/pharma-biopharma/drug-discovery-development/screening-compounds-libraries-hit-identification.html, accessed in April 2021). All the compounds were dissolved in DMSO, aliquoted, and stored at −80 °C until further use.

### 4.2. Dataset Assembly

The dataset assembly was based on a literature search of D_2_R ligands in the ChEMBL databank. Using ChEMBL, a set of actives (entries shown in Figure 3 and Table S1), inactives, and decoys was generated. The dataset was specifically filtered for D_2_R agonists with binding affinities of up to 500 nM. During dataset assembly, each ChEMBL entry and its corresponding publication was investigated considering binding affinity, methodological approach, and determination of D_2_R agonism. Finally, 68 ChEMBL entries including nine approved D_2_R agonists with binding affinity ranging from 0.075 nM up to 338 nM were used. The set of active compounds only compiled compounds with biological activities investigated in vitro. The set of inactives included 68 entries with binding affinities above 50 µM. The decoy dataset was generated utilizing the DUD-E webtool (http://dude.docking.org/, accessed on 15 March 2021) to create 3752 decoys based on the actives set. DS was used to ensure correctness of conformations, add hydrogens to the structures, and minimize the datasets. Datasets were generated using the “BEST settings”, with a maximum of 255 conformations per molecule in DS.

In LS screening, datasets were generated using the “iCon [112]-best” settings with a maximum of 200 conformations per molecule.

### 4.3. Generation of Pharmacophore Models

#### 4.3.1. SB Pharmacophore

SB pharmacophore models were based on the cryo-EM structure of D_2_R bound to agonist **2** (PDB ID 7jvr). In LS, a pharmacophore was created and transferred into the screening perspective. The original pharmacophore model was edited in a step-wise manner by altering feature tolerance and creation of new XVOLs. The different models were used to screen actives, inactives and decoy datasets maximizing recognition of actives while minimizing recognition of inactives and decoys. The pharmacophore model generated in DS was optimized using the same workflow.

#### 4.3.2. LB Pharmacophore

LB pharmacophore models generated in LS were based on the active compounds previously not identified by the LS SB model. Compounds **1**, **SC52**, and **SC59** were used to generate a shared feature pharmacophore model. Conformers were generated using the standard BEST setting. The resulting pharmacophore models were then optimized in the same way as the SB LS model. The generation of the LB model in DS was based on the same compounds as the LS model to increase comparability. The pharmacophore model generated in DS was optimized using the same workflow.

#### 4.3.3. Pharmacophore Model Validation Metrics

The quality of pharmacophore models is described by several parameters [113]. The most frequent are sensitivity (Equation (1)) and specificity (Equation (2)). Sensitivity expresses the probability of a randomly selected active compound to map the model. Specificity, on the other hand, describes the probability that a randomly selected inactive compound will not map the model.

Sensitivity = number of actives identified by the model/number of actives in the dataset(1)

Specificity = number of actives not identified by the model/number of inactives in the dataset(2)

Additionally, pharmacophore models are frequently described by parameters such as yield of actives (YoA), enrichment factor (EF), and accuracy. YoA (Equation (3)) specifies the ratio of true positives identified by the model to the number of all the compounds that mapped the model. EF (Equation (4)) as opposed to yield of actives also takes into account database sizes of active and inactive compounds, as well as of decoys. Accuracy refers to the probability of any compound being correctly classified either as active or inactive by the model (Equation (5)).

YoA = number of true positives/number of total hits(3)

EF = YoA/(number of actives in the database/number of all the compounds in the database)(4)

Accuracy = (number of true positives + number of true negatives)/number of all the compounds in the database(5)

Eventually, EF and YoA were utilized for evaluating the optimization process.

### 4.4. Predictive Screening—SPECS and Maybridge

SPECS (Specs_SC_10mg_Apr2021; 208,968 compounds) and Maybridge (MayBridge HitDiscover database; downloaded in April 2021, 51,775 compounds) databases were screened in the respective software used for the generation of the pharmacophore models. After the screening, the resulting virtual hits were processed in PipelinePilot applying a PAINS and Ro5-based physicochemical descriptor filter (Table 6). The limits for the physicochemical parameters were based on a detailed structural analysis of all the active compounds (compounds **1**–**9** and **SC1**–**SC59**) utilized during the pharmacophore model generation. Regarding PAINS, three different filters, PAINS1, 2, and 3, were applied using default settings [98,99]. PAINS filtering was followed by physicochemical descriptor filtering applying the criteria shown in Table 6.

Virtual hits meeting the PAINS and physicochemical descriptor criteria were further considered regarding ligand selection and in vitro investigation.

### 4.5. Settings for an HTRF-Compatible Tecan Spark Plate Reader

All the HTRF assays were performed using a Tecan Spark plate reader. The respective settings were specifically modified and optimized for the determination of D_2_R ligand-binding affinities. Experiments were performed using two different emission wavelengths at 620 and 665 nm, respectively, subsequently used for quantifying binding affinities. Fluorophores were excited at 320 nm. A Dichroic 510 mirror was used, while lag and integration times of 100 and 400 µs were applied, respectively. Flashes were set to 75. Electronic gain was automatically optimized, while the z-position was optimized based on the well with the highest expected signal. Experiments described in 4.6, 4.8, and 4.10 require the use of two 96-well plates. The first plate was used to determine the gain and the z-position. Subsequently, the determined values were set manually for the second plate to enable direct comparison between the different plates.

### 4.6. Characterization of D_2_R Carrier Cells—KD Determination

The cells used for the subsequent screening and detailed investigation of D_2_R ligands were acquired from PerkinElmer/cisbio (Tag-lite Dopamine D2-labeled Cells, ready-to-use (transformed and labeled), 200 tests; C1TT1D2). The cells were stored in liquid nitrogen until further use. Fluorescent ligand (Dopamine D2 Receptor red antagonist Fluorescent Ligand, stored at −20 °C; L0002RED), assay buffer (Tag-lite Buffer (5× concentrate), 100 mL, stored at 4 °C; LABMED), and 96-well plates (HTRF 96-well low-volume white plate; 66PL96005) required for the in vitro assay were also acquired from PerkinElmer/cisbio. Bromocriptine mesylate (50 mg; CAYM14598-50) was acquired from VWR. The assay was conducted according to the standard operation protocol (SOP) available from PerkinElmer/cisbio. The 96-well plates were incubated at room temperature for 2 h. The 96-well plates were read on a Tecan Spark plate reader (the settings are shown in Section 4.5). The respective concentrations of the dilution series were performed in triplicates. In total, K_D_ determination was performed three times.

### 4.7. Compound Selection for Assessing In Vitro Activity

Ligand selection regarding activity screening was performed using fitness score, scaffold similarity, and model origin as a selection matrix. Thus, ligands found by the different pharmacophore models (consensus hits) were prioritized. Consensus hits were categorized as the hits found by M2 + M4 + M1/M3 (triple consensus), M2 + M4, M2 + M1/M3, or M4 + M1/M3 (double consensus). Single hits originating from only one of the pharmacophore models were selected in a balanced manner to enable validation of the respective models later on. Structural diversity was assessed using DataWarrior [114] based on linker regions, ring sizes, number of rings, and functional groups. Considering similar compounds, compounds with lower fitness scores were excluded from the selection.

### 4.8. In Vitro Assessment of Compound Activity

Materials described in 4.6 were also used during ligand screening. TLB (1×) was prepared as described earlier. For ligand screening, compounds were prepared at a working solution concentration of 40 µM in 1× TLB. Known D_2_R ligands (both agonists and antagonists) were used as the positive controls at the same concentration. The assay was conducted according to the SOP available from PerkinElmer/cisbio. The 96-well plates were incubated at room temperature in the dark for 2 h. The 96-well plates were read on a Tecan Spark plate reader (the settings are shown in Section 4.5). In total, ligand screening was performed twice.

### 4.9. Ligand Selection for Detailed In Vitro Investigation (K_I_ Determination)

**Figure 10:**
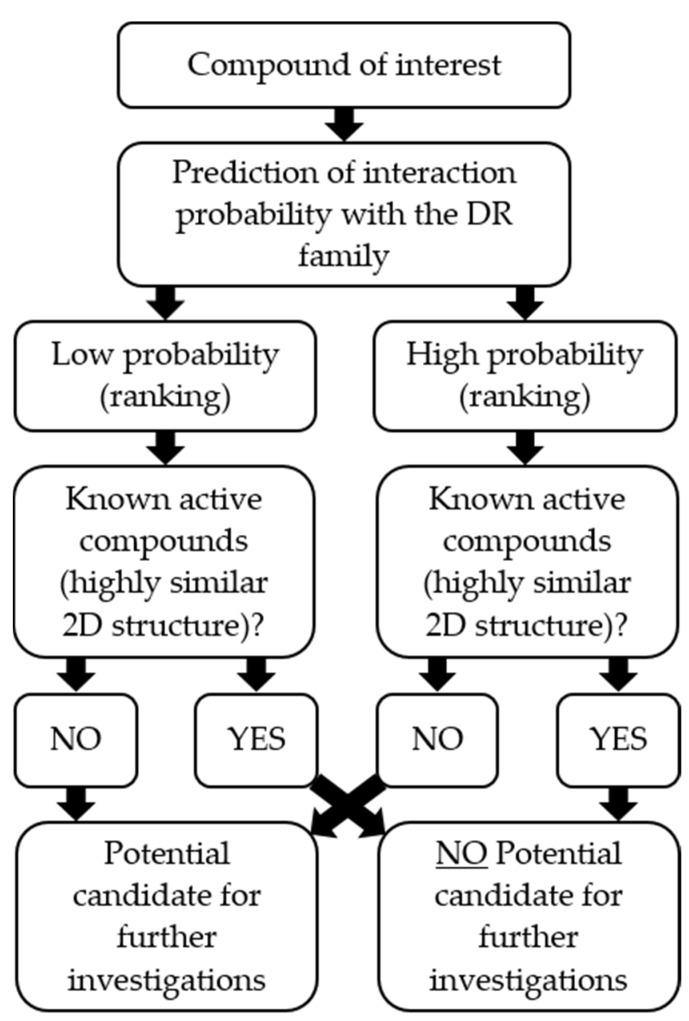
Decision tree following the investigation of the compounds of interest based on a SwissTargetPrediction search.

Ligands were considered active when they had a DNF of ≥ 2 compared to the control. Active ligands were investigated using a literature cross-reference search (SciFinder) and predicting the compounds’ most likely molecular targets (SwissTargetPrediction). In SciFinder, a database of all publications based on the search phrase “dopamine receptor” was generated. Active ligands were cross-referenced using their exact structure as well as similar structures (similarity score of ≥80) with the literature database. Exact structural matches with a known activity at D_2_R were automatically excluded from the selection. Similarity matches were considered as candidates. Swiss target prediction was used to refine compound selection based on the SciFinder search. Therefore, compounds of interest were investigated considering their probability to interact with the DR family as well as the availability of structurally similar compounds (focusing on 2D structures). The results from the Swiss target prediction analysis were evaluated based on the decision tree shown in Figure 10.

Finally, the candidates were sorted by activity and investigated considering scaffold diversity.

### 4.10. K_I_ Determination of the Selected Ligands

The materials described in 4.6 were also used during K_I_ determination of the ligands selected after screening. The selected ligands (compounds **14**, **15**, **16**, **17**, **18**, and **19**) were diluted in 1× TLB. Compounds **14**, **15**, **18**, and **19** were diluted to an initial working solution concentration of 4 × 10^−4^ M. Compound **15** was diluted to an initial working solution concentration of 4 × 10^−5^ M. Compound **16** was diluted to an initial working solution concentration of 1 × 10^−4^ M. Different concentrations were chosen due to differences in aqueous solubility of the compounds. K_I_ was determined following the SOP available at PerkinElmer/cisbio. The 96-well plates were incubated at room temperature in the dark for 2 h. The 96-well plates were read on a Tecan Spark plate reader (the settings are shown in Section 4.5). In total, K_I_ determination was performed twice.

### 4.11. Pharmacophore Model Evaluation

The theoretical model validation was based on the metrics described in Section 4.3.3. The prospective performance was evaluated based on the models’ predictive power identifying previously unknown D_2_R ligands. It was quantified as the hit rate (%).

### 4.12. Assessing Scaffold Similarity/Dissimilarity

Canonical SMILES codes of all the compounds of interest were imported to Canvas 3.8. In Canvas 3.8, radial fingerprints (ECFP4 [115,116]) of all the molecules (based on 2D structures) were calculated followed by an automated calculation of a TS [100] for each compared pair. TS matrices were exported to Excel as .csv files and imported to GraphPad Prism 8 to display heatmaps, color-coding the structural similarities. TSs range from 0 to 1. An increasing coefficient indicated an increasing structural similarity. In each similarity assessment, the 2D structures of the most similar compound pairs were observed in detail to investigate compound novelty and scaffold diversity.

### 4.13. Data Processing, Representation, and Analysis

Pharmacophore modelling and virtual screening were performed in LS version 4.4.5 (Inte:Ligand GmbH, Vienna, Austria) and DS 2018 (DS, Accelrys Inc., San Diego, CA, USA). Two-dimensional structures of all the compounds were generated in ChemDraw 19.0 (PerkinElmer, Waltham, MA, USA). Sd files used for similarity assessments were generated in PipelinePilot Client 9.1 [95] and processed in Canvas 3.8 (Canvas, Schrödinger, LLC, New York, NY, USA, 2021) and DataWarrior [114]. Heatmaps, NDF, and saturation curves were processed and visualized in GraphPad Prism 8.

## Figures and Tables

**Table 1:** Theoretical evaluation of the generated pharmacophore models. The models were evaluated and compared to each other regarding model accuracy, enrichment factor (EF), and yield of actives (YoA). TPs, true positives. FPs, false positives. TNs, true negatives.

	M1	M2	M3	M4	All Models
Active hits (n = 68)	9	16	26	30	51
Inactive hits (n = 68)	1	0	1	0	2
Decoy hits (n = 3752)	35	36	9	27	108
TPs	9	16	26	30	51
FPs	36	36	10	27	110
TNs	3784	3783	3810	3792	3710
Specificity	0.99	0.99	1.00	0.99	0.97
Sensitivity	0.13	0.24	0.38	0.44	0.75
Accuracy	0.98	0.98	0.99	0.98	0.97
EF	11.44	17.26	41.29	29.57	18.11
YoA	0.20	0.30	0.74	0.52	0.32

**Table 2:** Summary of virtual hits after screening and filtering of the SPECS and Maybridge databases. The SPECS and Maybridge databases were screened separately with the four individual pharmacophore models generated in DS and LS. Double and triple consensus hits refer to different combinations of virtual hits from the single-hit groups.

		Number of Hits		
Pharmacophore Model		SPECS	Maybridge	Combined
Single hits	M1	236	118	354
	M2	595	76	671
	M3	157	17	174
	M4	762	78	840
Consensus hits (n = 2)	M2 + M4	122	11	133
	M1 + M3	0	0	0
	M4 + M1/M3	47	6	53
	M2 + M1/M3	10	1	11
Consensus hits (n = 3)	M2 + M4 + M1/M3	4	0	4

**Table 3:** Summary of the in vitro binding assessment of the compounds considered active. All measurements were conducted at a concentration of 10 µM (n = 4). Fluorescence decrease was normalized to the control.

Compound	Normalized Decrease in Fluorescence ± SD	Compound	Normalized Decrease in Fluorescence ± SD
Control	1	**SC160**	2.41 ± 2.01
**1**	9.44 ± 5.97	**15**	3.99 ± 2.58
Dopamine	1.50 ± 0.88	**SC175**	2.98 ± 1.77
Sulpiride	30.65 ± 6.79	**16**	15.74 ± 18.15
**2**	39.10 ± 1.31	**17**	8.18 ± 3.62
Droperidol	38.69 ± 2.56	**SC191**	1.83 ± 0.83
Haloperidol	39.18 ± 0.83	**18**	10.85 ± 4.93
Piribedil	9.21 ± 3.80	**SC198**	2.43 ± 1.39
**14**	40.40 ± 1.39	**SC201**	1.99 ± 0.85
**SC149**	2.04 ± 1.34	**19**	22.08 ± 6.62
**SC155**	1.68 ± 0.90	**SC207**	22.89 ± 8.41

**Table 4:** Summary of the determined activities: K_I_ values of the different compounds at D_2_R. Activities are also expressed as fold differences in comparison to the determined K_I_ of dopamine. K_I_ values in µM were determined using n = 6; 95% confidence intervals (CI) are shown. Dopamine is shown as the control.

Compound No. (ID)	Fold Difference	K_I_ (µM)	95% CI (µM)
**14**	2700	0.004	0.003–0.006
**15**	2.6	4.31	2.01–19.58
**16**	1.1	10.04	5.69–17.43
**17**	9.0	1.24	0.77–2.05
**18**	4.2	2.63	1.62–4.28
**19**	34.7	0.32	0.23–0.45
Dopamine	1	11.12	7.77–15.84

**Table 5:** Prospective performance of the pharmacophore models generated in DS and LS.

D_2_R Ligands (Identified by Models)	Tested Virtual Hits	Active Virtual Hits	Number of Active Hits	Hit Rate (%)
M1	22	**17**	1	4.5
M2	37	**15**, **16**, **17**, **18**	4	10.8
M3	25	**14**, **15**, **19**	3	12
M4	45	**14**, **16**, **19**	3	6.7

**Table 6:** Summary of the parameter restrictions applied during physicochemical descriptor filtering prior to virtual hit selection.

Parameter	Value
Molecular weight (MW) (g/mol)	600 ≥ x ≥ 200
cLogP	6.0 ≥ x ≥ 1.5
HB acceptors	7 ≥ x ≥ 0
HB donors	3 ≥ x ≥ 0
Rotatable bonds	18 ≥ x ≥ 0
Rings	5 ≥ x ≥ 2
Aromatic rings	3 ≥ x ≥ 1

## Data Availability

The data presented in this study are available in Supplementary Material. If further data are required, they are available from the corresponding author upon request.

## Supplementary Materials

The following are available online at https://www.mdpi.com/article/10.3390/molecules27144435/s1, Table S1: Dataset of active compounds, Table S2: Dataset of inactives, Table S3: Evaluation of the optimization process of model M1, Table S4: Evaluation of the optimization process of model M2, Table S5: Evaluation of the optimization process of model M3, Table S6: Evaluation of the optimization process of model M4, Table S7: Selection of virtual hits after virtual screening, Table S8: Similarity assessment comparing the selected virtual hits, Table S9: Summary of the compounds selected for further in vitro investigation, Table S10: Overview of in vitro activity screening of all the selected virtual hits, Table S11: Similarity assessment comparing the identified ligands and the dataset of actives, Table S12: Assessment of ligand novelty compared to ChEMBL entries, Figure S1: Original SB pharmacophore models, Figure S2: Active compounds used for LB pharmacophore approaches, Figure S3: Original LB pharmacophore models, Figure S4: Alignment of LB models with **SC59**, Figure S5: Overview of similarity assessments in single- and consensus-hit groups, Figure S6: Overview of in vitro activity screening of all the selected virtual hits, Figure S7: Overview of K_I_ values of the identified ligands, Figure S8: Assessment of scaffold diversity comparing the identified ligands and the dataset of actives, Figure S9: Alignment of compound **15** with M2 and M3, Figure S10: Alignment of compound **16** with M2 and M4, Figure S11: Alignment of compound **17** with M1 and M2, Figure S12: Alignment of compound **18** with M2, Figure S13: Alignment of compound **19** with M3 and M4.

## Sample Availability

Samples of the compounds are not available from the authors.
