# Supplementary material for: Identification of Novel Dopamine D2 Receptor Ligands—A Combined In Silico/In Vitro Approach
Source: Molecules. 2022 Jul 11;27(14):4435. doi: 10.3390/molecules27144435 (PMC9318694; doi:10.3390/molecules27144435)
Supplement: Supplementary file 1 [file molecules-27-04435-s001.zip › molecules-1757763-supplementary.pdf]

Table of content – supplementary information

1. Overview of D_2R 3D structures entered into PDB 1
2. Data set assembly 1
2.1. Data set of actives 1
2.2. Data set of inactives 5
3. Theoretical evaluation of generated pharmacophore models 9
3.1. M1 – Structure-based LigandScout model 9
3.2. M2 – Structure-based DiscoveryStudio model 10
3.3. M3 – Ligand-based LigandScout model 11
3.4. M4 – Ligand-based DiscoveryStudio model 12
4. Selection of compounds after virtual screening – allocation to the different pharmacophore models 13
5. Similarity assessment – comparison of compounds from virtual hit groups 14
6. Hit selection after virtual screening – overview of 2D structures and NDF values of all compounds 15
7. Compound selection after in vitro screening – literature analysis 21
8. Similarity assessment – investigation of identified D_2R ligands 22
8.1. Comparison to data set of actives 22
8.2. Comparison to ChEMBL 23
9. Pharmacophore models 24
9.1. Display of the original structure-based pharmacophore models M1 and M2 24
9.2. Selected compounds from the data set of actives – generation of ligand-based pharmacophore models M3 and M4 24
9.3. Display of the original ligand-based pharmacophore models M3 and M4 25
9.4. Assessing comparability of M3 and M4 based on feature location superimposed with compound SC59 25
10. Tanimoto score matrices – similarity assessments in the different virtual hit groups 25
11. In vitro analysis of selected virtual hits 27
11.1. Summary of in vitro activity (based on NDF values) of control compounds and selected virtual hits 27
11.2. Summary of K_I values determined in vitro – overview of identified D_2R ligands 28
12. Assessing scaffold diversity – novel ligands vs. active data set 28
13. Identified D_2R ligands – pharmacophore model alignments 29

1. Overview of D_2R 3D structures entered into PDB

The crystal structure of D_2R bound to risperidone (PDB ID 6cm4) tried to shed light on D_2R ligand agonism and antagonism, highlighting determining structural elements during D_2R receptor (in-)activation. The co-crystallization of spiperone (PDB ID 7dfp) and haloperidol (PDB ID 6luq) support the search for truly selective D_2R ligands based on structural elements of spiperone and haloperidol contributing to D_2R-selectivity. Recently, two different agonist-bound D_2R cryo-EM structures were published. Both PDB entries (6vms and 7jvr) are bound to bromocriptine and elucidate agonist binding interactions.

2. Data set assembly

2.1. Data set of actives

Compounds included both approved D_2R agonists (compounds **1** to **9**) as well as research compounds (**SC1** to **SC59**).

Table S1. Data set used for the generation and training of pharmacophore models in LigandScout and DiscoveryStudio. Highly active, known D_2R agonists were chosen as training molecules based on a ChEMBL literature search.

ID	Compound name	K_I [nM][1]	Reference
**1**	Apomorphine	1.8	[50]
**2**	Bromocriptine	0.9	[51]
**3**	Cabergoline	0.7	[52]
**4**	Lisuride	0.66	[53]
**5**	Pergolide	3.4	[54]
**6**	Pramipexole	3.3	[55]
**7**	Ropinirole	7.2	[56]
**8**	Rotigotine	0.06	[57]
**9**	Sumanirole	46.3	[58]
**SC1**	(R)-2-((benzylamino)methyl)chroman-7-ol	2.98	[59]
**SC2**	4,4'-(((octane-1,8-diylbis(1H-1,2,3-triazole-1,4-diyl))bis(propane-3,1-diyl))bis(oxy))bis(N-(4-((2,3-dihydro-1H-inden-2-yl)(propyl)amino)butyl)-3-methoxybenzamide)	16	[60]
**SC3**	4-(3-(1-butyl-1H-1,2,3-triazol-4-yl)propoxy)-N-(4-((2,3-dihydro-1H-inden-2-yl)(propyl)amino)butyl)-3-methoxybenzamide	12	
**SC4**	5-(4-(4-(2,3-dichlorophenyl)piperidin-1-yl)butoxy)-1,3-dihydro-2H-benzo[d]imidazol-2-one	11	[61]
**SC5**	7-(3-(4-(2,3-dichlorophenyl)piperazin-1-yl)propoxy)-3,4-dihydroisoquinolin-1(2H)-one	18	
**SC6**	5-(4-(4-(2,3-dichlorophenyl)piperidin-1-yl)butoxy)benzo[d]thiazole	42	[61]
**SC7**	5-(3-(4-(2,3-dichlorophenyl)piperidin-1-yl)propoxy)benzo[d]thiazole	75	
**SC8**	5-(3-(4-(2,3-dichlorophenyl)piperazin-1-yl)propoxy)benzo[d]thiazole	30	
**SC9**	5-(3-(4-(2,3-dichlorophenyl)-1,4-diazepan-1-yl)propoxy)benzo[d]thiazole	20	

ID	Compound name	K_I [nM][1]	Reference
**SC10**	(S)-N-(4-((4-ethynylcyclohex-3-en-1-yl)(propyl)amino)butyl)-[1,1'-biphenyl]-4-carboxamide	9.4	[62]
**SC11**	(R)-N-(4-((4-ethynylcyclohex-3-en-1-yl)(propyl)amino)butyl)-[1,1'-biphenyl]-4-carboxamide	14	
**SC12**	(R)-N-(4-((4-ethynylcyclohex-3-en-1-yl)(propyl)amino)butyl)pyrazolo[1,5-a]pyridine-2-carboxamide	5.5	[63]
**SC13**	(R)-N-(4-((4-ethynylcyclohex-3-en-1-yl)(propyl)amino)butyl)pyrazolo[1,5-a]pyridine-3-carboxamide	1.1	
**SC14**	(R)-4-(3-(2-butyl-2H-1,2,3-triazol-4-yl)propoxy)-N-(4-((4-ethynylcyclohex-3-en-1-yl)(propyl)amino)butyl)-3-methoxybenzamide	4.2	
**SC15**	(R)-N-(4-((4-ethynylcyclohex-3-en-1-yl)(propyl)amino)butyl)benzo[b]thiophene-2-carboxamide	3.1	
**SC16**	Quinpirole	6.4	[64]
**SC17**	(R)-5-(dipropylamino)-5,6-dihydro-4H-imidazo[4,5,1-ij]quinolin-2-ol	11.4	[70]
**SC18**	5-OH-DPAT	58.8	[65]
**SC19**	7-OH-DPAT	202	
**SC20**	(R)-2-(((4-iodobenzyl)amino)methyl)chroman-7-ol	3.79	[59]
**SC21**	(R)-2-(((3-iodobenzyl)amino)methyl)chroman-7-ol	2.06	
**SC22**	(R)-2-(((4-(4-fluorobutoxy)benzyl)amino)methyl)chroman-7-ol	5.6	
**SC23**	(R)-2-(((2-(4-(4-(2-fluoroethoxy)phenyl)piperazin-1-yl)ethyl)amino)methyl)chroman-7-ol	6.67	
**SC24**	(R)-2-((hexylamino)methyl)chroman-7-ol	8.19	
**SC25**	8-isopentyl-6,6a,7,8,9,10-hexahydro-4H-pyrazino[1,2-a]pyrrolo[4,3,2-de]quinoline	24	[53]
**SC26**	8-(cyclohexylmethyl)-6,6a,7,8,9,10-hexahydro-4H-pyrazino[1,2-a]pyrrolo[4,3,2-de]quinoline	53.7	
**SC27**	3-amino-5-chloro-6-methoxy-N-(5-(4-(2-methoxyphenyl)piperazin-1-yl)pentyl)-4-methylthieno[2,3-b]pyridine-2-carboxamide	3.5	[66]
**SC28**	tert-butyl (5-(4-(2,3-dichlorophenyl)piperazin-1-yl)pentyl)carbamate	42.6	
**SC29**	3-amino-N-(5-(4-(2,3-dichlorophenyl)piperazin-1-yl)pentyl)-4,6-dimethylthieno[2,3-b]pyridine-2-carboxamide	11.2	

ID	Compound name	K$_I$ [nM][1]	Reference
**SC30**	tert-butyl (3-(4-(2-methoxyphenyl)piperazin-1-yl)propyl)carbamate	15.85	
**SC31**	tert-butyl (5-(4-(2-methoxyphenyl)piperazin-1-yl)pentyl)carbamate	12.02	
**SC32**	(R)-4-phenyl-1-((2,3,4,5-tetrahydro-[1,1'-biphenyl]-3-yl)methyl)-1,2,3,6-tetrahydropyridine	8.4	[67]
**SC33**	(R)-3'-((4-phenyl-3,6-dihydropyridin-1(2H)-yl)methyl)-2',3',4',5'-tetrahydro-[1,1'-biphenyl]-4-ol	0.62	
**SC34**	(R)-4-(3-(1-butyl-1H-1,2,3-triazol-4-yl)propoxy)-N-(4-((6-ethynyl-3,4-dihydro-2H-pyran-3-yl)(propyl)amino)butyl)-3-methoxybenzamide	1.6	[62]
**SC35**	(S)-4-(3-(1-butyl-1H-1,2,3-triazol-4-yl)propoxy)-N-(4-((6-ethynyl-3,4-dihydro-2H-pyran-3-yl)(propyl)amino)butyl)-3-methoxybenzamide	4.9	
**SC36**	4-(3-(1-butyl-1H-1,2,3-triazol-4-yl)propoxy)-N-(4-((2-(8-hydroxy-2-oxo-1,2-dihydroquinolin-5-yl)ethyl)(propyl)amino)butyl)-3-methoxybenzamide	24	
**SC37**	(S)-4-(3-(1-butyl-1H-1,2,3-triazol-4-yl)propoxy)-N-(4-((5-hydroxy-1,2,3,4-tetrahydronaphthalen-2-yl)(propyl)amino)butyl)-3-methoxybenzamide	0.47	
**SC38**	(R)-5-(dipropylamino)-1-propyl-5,6-dihydro-4H-imidazo[4,5,1-ij]quinolin-2(1H)-one	2.59	[70]
**SC39**	(R)-1-butyl-5-(methylamino)-5,6-dihydro-4H-imidazo[4,5,1-ij]quinolin-2(1H)-one	12.8	
**SC40**	(R)-5-(methylamino)-1-propyl-5,6-dihydro-4H-imidazo[4,5,1-ij]quinolin-2(1H)-one	13.2	
**SC41**	(R)-1-propyl-5-(propylamino)-5,6-dihydro-4H-imidazo[4,5,1-ij]quinolin-2(1H)-one	8.08	
**SC42**	(R)-1-(4-(4-phenylbutoxy)butyl)-5-(propylamino)-5,6-dihydro-4H-imidazo[4,5,1-ij]quinolin-2(1H)-one	37.2	[58]
**SC43**	(R)-N-(4-(5-(dipropylamino)-2-oxo-5,6-dihydro-4H-imidazo[4,5,1-ij]quinolin-1(2H)-yl)butyl)-1H-indole-2-carboxamide	86.8	
**SC44**	(R)-5-(methylamino)-1-(4-((2-oxo-1,2,3,4-tetrahydroquinolin-7-yl)oxy)butyl)-5,6-dihydro-4H-imidazo[4,5,1-ij]quinolin-2(1H)-one	338	
**SC45**	(R)-5-(methylamino)-1-(2-(2-(2-(4-phenylbutoxy)ethoxy)ethoxy)ethyl)-5,6-dihydro-4H-imidazo[4,5,1-ij]quinolin-2(1H)-one	34.7	
**SC46**	8-hydroxy-5-(4-(4-(pyrazolo[1,5-a]pyridin-5-yloxy)butyl)-1,4-diazepan-1-yl)quinolin-2(1H)-one	78	[68]
**SC47**	(R)-N-(4-((2-oxo-1-propyl-1,2,5,6-tetrahydro-4H-imidazo[4,5,1-ij]quinolin-5-	8.43	[58]

ID	Compound name	K_I [nM][1]	Reference
	yl)(propyl)amino)butyl)-1H-indole-2-carboxamide		
**SC48**	(R)-5-(methylamino)-1-(2-(2-(2-(2-((2-oxo-1,2,3,4-tetrahydroquinolin-7-yl)oxy)ethoxy)ethoxy)ethoxy)ethyl)-5,6-dihydro-4H-imidazo[4,5,1-ij]quinolin-2(1H)-one	306	
**SC49**	(R)-5-(methyl(4-((2-oxo-1,2,3,4-tetrahydroquinolin-7-yl)oxy)butyl)amino)-5,6-dihydro-4H-imidazo[4,5,1-ij]quinolin-2(1H)-one	40.1	
**SC50**	(R)-5-(methylamino)-1-(2-(2-(4-phenylbutoxy)ethoxy)ethyl)-5,6-dihydro-4H-imidazo[4,5,1-ij]quinolin-2(1H)-one	111	
**SC51**	(R)-5-(methylamino)-1-(12-((2-oxo-1,2,3,4-tetrahydroquinolin-7-yl)oxy)dodecyl)-5,6-dihydro-4H-imidazo[4,5,1-ij]quinolin-2(1H)-one	284	
**SC52**	8-hydroxy-5-(4-(4-((2-oxo-1,2,3,4-tetrahydroquinolin-7-yl)oxy)butyl)-1,4-diazepan-1-yl)quinolin-2(1H)-one	1.6	[68]
**SC53**	(S)-N6-(2-(4-([1,1'-biphenyl]-4-yl)piperazin-1-yl)ethyl)-N6-propyl-4,5,6,7-tetrahydrobenzo[d]thiazole-2,6-diamine	264	[65]
**SC54**	(R)-6-propyl-5,6,6a,7-tetrahydro-4H-dibenzo[de,g]quinoline-10,11-diol	0.075	[59]
**SC55**	(S)-N6-propyl-N6-(2-(4-(quinolin-5-yl)piperazin-1-yl)ethyl)-4,5,6,7-tetrahydrobenzo[d]thiazole-2,6-diamine	57.7	
**SC56**	(S)-N6-(2-(4-(isoquinolin-1-yl)piperazin-1-yl)ethyl)-N6-propyl-4,5,6,7-tetrahydrobenzo[d]thiazole-2,6-diamine	269	[65]
**SC57**	(S)-N6-propyl-N6-(2-(4-(quinolin-4-yl)piperazin-1-yl)ethyl)-4,5,6,7-tetrahydrobenzo[d]thiazole-2,6-diamine	109	
**SC58**	4-(4-(2-((5-hydroxy-1,2,3,4-tetrahydronaphthalen-2-yl)(propyl)amino)ethyl)piperazin-1-yl)quinolin-8-ol	3.75	[69]
**SC59**	Naxagolide	0.45	[59]

[1] All K_I values retrieved from ChEMBL were determined in vitro utilizing radio-ligand binding assays.

2.2. *Data set of inactives*

Table S2. Overview of the inactives data set retrieved from ChEMBL. Compounds were considered inactive with a $K_I > 50$ µM. Compounds were only included in the dataset if biological activities were determined in vitro. All K_I values were determined using radio-ligand binding (RLB) assays.

ID	Compound name	KI [µM]²	Reference
**SC60**	3-[[4-(2-methoxyphenyl)piperazin-1-yl]methyl]-2,7-dimethylimidazo[1,2-c]pyrimidine	53	[71]
**SC61**	5-phenyl-2,3,4,5-tetrahydro-1H-3-benzazepine-7,8-diol	720	[72]
**SC62**	N-[4-[4-(4-chlorophenyl)piperazin-1-yl]butyl]-5-phenyl-1,2,4-oxadiazol-3-amine;hydrochloride	79	[73]
**SC63**	N-(3,4a,9,9a-tetrahydro-2H-indeno[1,2-b][1,4]dithiin-6-yl)-1-(6,7-dimethoxy-3,4-dihydro-1H-isoquinolin-2-yl)methanimine	>100	[74]
**SC64**	N-(2,3,4a,5,6,10b-hexahydrobenzo[f][1,4]benzodithiin-9-yl)-1-(6,7-dimethoxy-3,4-dihydro-1H-isoquinolin-2-yl)methanimine	>300	
**SC65**	6-methoxy-1-(1-methyl-4-nitropyrrol-2-yl)-4,9-dihydro-3H-pyrido[3,4-b]indole	200	
**SC66**	N-(2,3,4a,5,6,10b-hexahydrobenzo[f][1,4]benzodithiin-9-yl)-1-(6,7-dimethoxy-3,4-dihydro-1H-isoquinolin-2-yl)methanimine	>100	
**SC67**	6-[(6,7-dimethoxy-3,4-dihydro-1H-isoquinolin-2-yl)methylideneamino]-2,3-dihydro-1H-inden-1-ol	>300	
**S68**	6-(3,4-dihydro-1H-isoquinolin-2-ylmethylideneamino)-2,3-dihydro-1H-inden-1-ol	>300	
**SC69**	4-[(2-chlorophenyl)sulfonylmethyl]-6-(4-methylpiperazin-1-yl)-1,3,5-triazin-2-amine	50,141	[75]
**SC70**	[3-[[4-(4-chlorophenyl)piperazin-1-yl]methyl]pyrazolo[1,5-a]pyridin-4-yl]methanol	79	[76]
**SC71**	2-[[5-[(4-phenylpiperazin-1-yl)methyl]-1H-pyrrol-2-yl]methylidene]propanedinitrile	>100	[77]
**SC72**	2-[(R)-benzhydrylsulfinyl]acetamide	>100	[78]
**SC73**	N-propan-2-yl-1H-indole-2-carboxamide	>100	[79]
**SC74**	1,2,3,4-tetrahydroisoquinoline-6-carbonitrile	53,8	
**SC75**	N-(cyclopropylmethyl)-1H-indole-2-carboxamide	>100	
**SC76**	1-(2-methoxyphenyl)-6-[3-[(4-methyl-5-phenyl-1,2,4-triazol-3-yl)sulfanyl]propyl]-1,6-diazaspiro[3.3]heptane	114,945	[92]
**SC77**	1-(4-fluorophenyl)-6-[3-[(4-methyl-5-phenyl-1,2,4-triazol-3-yl)sulfanyl]propyl]-1,6-diazaspiro[3.3]heptane	>109,574	
**SC78**	3-(4-methoxyphenyl)-9-[3-[(4-methyl-5-phenyl-1,2,4-triazol-3-yl)sulfanyl]propyl]-3,9-diazaspiro[5.5]undecane	104,847	

ID	Compound name	KI [µM]²	Reference
**SC79**	2-[4-(4-chlorophenyl)piperidin-1-yl]-N-(3-methylphenyl)acetamide	>50	[91]
**SC80**	N-(3-ethylphenyl)-2-(4-pyridin-2-ylpiperazin-1-yl)acetamide	>50	
**SC81**	2-(4-pyridin-2-ylpiperazin-1-yl)-N-pyrimidin-5-ylacetamide	>50	
**SC82**	2-[4-(5-chloropyridin-2-yl)piperazin-1-yl]-N-pyrimidin-5-ylacetamide	>50	
**SC83**	2-[4-(5-chloropyridin-2-yl)piperazin-1-yl]-N-(3-methylphenyl)acetamide	>50	
**SC84**	N-(3-methylphenyl)-2-[4-(5-methylpyridin-2-yl)piperidin-1-yl]acetamide	>50	
**SC85**	2-[4-(5-chloropyridin-2-yl)piperazin-1-yl]-N-pyridin-3-ylacetamide	>50	
**SC86**	N-pyridin-3-yl-2-(4-pyridin-2-ylpiperazin-1-yl)acetamide	>50	
**SC87**	3-[3-[(4-methyl-5-phenyl-1,2,4-triazol-3-yl)sulfanyl]propyl]-3,9-diazaspiro[5.5]undecane	>225,748	[80]
**SC88**	3-(2,4-dimethoxyphenyl)-9-[3-[(4-methyl-5-phenyl-1,2,4-triazol-3-yl)sulfanyl]propyl]-3,9-diazaspiro[5.5]undecane	65,229	
**SC89**	4,4-dimethyl-1-[3-[(4-methyl-5-phenyl-1,2,4-triazol-3-yl)sulfanyl]propyl]piperidine	59,994	
**SC90**	4-methyl-3-phenyl-5-propylsulfanyl-1,2,4-triazole	180,375	
**SC91**	1-[3-[(4-methyl-5-phenyl-1,2,4-triazol-3-yl)sulfanyl]propyl]piperazine	74,272	
**SC92**	N-[2-[(1S,2R)-2-[[(2S,5S)-2-(6-aminopyridin-3-yl)-5-methylmorpholin-4-yl]methyl]cyclopropyl]ethyl]-1H-indole-2-carboxamide	70,6	[81]
**SC93**	6,9-dichloro-3-methyl-5-phenyl-1,2,4,5-tetrahydro-3-benzazepine-7,8-diol;hydrochloride	810	[82]
**SC94**	methyl 5-(propylamino)-4,5,6,7-tetrahydropyrazolo[1,5-a]pyridine-3-carboxylate	>100	[83]
**SC95**	2-[[4-(4-fluorophenyl)piperazin-1-yl]methyl]imidazo[1,2-a]pyrazine	68	[84]
**SC96**	(4-bromothiophen-2-yl)-[4-fluoro-4-[[(5-methylpyridin-2-yl)methylamino]methyl]piperidin-1-yl]methanone	100	[85]
**SC97**	(5R)-N,N-dipropyl-4,5,6,7-tetrahydropyrazolo[1,5-a]pyridin-5-amine	55	[86]
**SC98**	3-[[4-[4-(2-fluoroethoxy)phenyl]piperazin-1-yl]methyl]pyrazolo[1,5-a]pyridine	79	[87]
**SC99**	4-phenyl-N-[4-(propylamino)butyl]benzamide	>100	[88]
**SC100**	1-(4-fluorophenyl)-4-[4-[2-[2-(3-methoxyphenyl)ethyl]phenoxy]butyl]piperazine	1324	[89]

ID	Compound name	KI [µM]²	Reference
**SC101**	2-[4-[2-[2-[2-(3-methoxyphenyl)ethyl]phenoxy]ethyl]piperazin-1-yl]pyrimidine	629	
**SC102**	1-(3-chlorophenyl)-4-[4-[2-[2-(3-methoxyphenyl)ethyl]phenoxy]butyl]piperazine	>1000	
**SC103**	1-(3-chlorophenyl)-4-[5-[2-[2-(3-methoxyphenyl)ethyl]phenoxy]pentyl]piperazine	>1000	
**SC104**	1-[2-[2-[2-(3-methoxyphenyl)ethyl]phenoxy]ethyl]-4-[3-(trifluoromethyl)phenyl]piperazine	>1000	
**SC105**	1-[3-[2-[2-(3-methoxyphenyl)ethyl]phenoxy]propyl]-4-[3-(trifluoromethyl)phenyl]piperazine	1412	
**SC106**	1-(4-fluorophenyl)-4-[3-[2-[2-(3-methoxyphenyl)ethyl]phenoxy]propyl]piperazine	655	
**SC107**	1-(3-chlorophenyl)-4-[3-[2-[2-(3-methoxyphenyl)ethyl]phenoxy]propyl]piperazine	298	
**SC108**	1-(2-methoxyphenyl)-4-[5-[2-[2-(3-methoxyphenyl)ethyl]phenoxy]pentyl]piperazine	>1000	
**SC109**	1-(4-fluorophenyl)-4-[2-[2-[2-(3-methoxyphenyl)ethyl]phenoxy]ethyl]piperazine	>1000	
**SC110**	1-[4-[2-[2-(3-methoxyphenyl)ethyl]phenoxy]butyl]-4-[3-(trifluoromethyl)phenyl]piperazine	>1000	
**SC111**	1-[5-[2-[2-(3-methoxyphenyl)ethyl]phenoxy]pentyl]-4-methylpiperazine	>1000	
**SC112**	6,7-dimethoxy-2-[4-[2-[2-(3-methoxyphenyl)ethyl]phenoxy]butyl]-3,4-dihydro-1H-isoquinoline	>1000	
**SC113**	2-[4-[2-[2-(3-methoxyphenyl)ethyl]phenoxy]butyl]-3,4-dihydro-1H-isoquinoline	>1000	
**SC114**	6,7-dimethoxy-2-[2-[2-[2-(3-methoxyphenyl)ethyl]phenoxy]ethyl]-3,4-dihydro-1H-isoquinoline	>1000	
**SC115**	6,7-dimethoxy-2-[3-[2-[2-(3-methoxyphenyl)ethyl]phenoxy]propyl]-3,4-dihydro-1H-isoquinoline	>1000	
**SC116**	1-[4-[2-[2-(3-methoxyphenyl)ethyl]phenoxy]butyl]-4-methylpiperazine	>1000	
**SC117**	2-[4-[4-[2-[2-(3-methoxyphenyl)ethyl]phenoxy]butyl]piperazin-1-yl]pyrimidine	854	
**SC118**	2-[4-[5-[2-[2-(3-methoxyphenyl)ethyl]phenoxy]pentyl]piperazin-1-yl]pyrimidine	>1000	

ID	Compound name	KI [μM][2]	Reference
**SC119**	1-(4-fluorophenyl)-4-[5-[2-[2-(3-methoxyphenyl)ethyl]phenoxy]pentyl]piperazine	847	
**SC120**	1-(2-methoxyphenyl)-4-[2-[2-[2-(3-methoxyphenyl)ethyl]phenoxy]ethyl]piperazine	427	
**SC121**	1-(3-chlorophenyl)-4-[2-[2-[2-(3-methoxyphenyl)ethyl]phenoxy]ethyl]piperazine	>1000	
**SC122**	1-(2-methoxyphenyl)-4-[4-[2-[2-(3-methoxyphenyl)ethyl]phenoxy]butyl]piperazine	67	
**SC123**	1-[3-[2-[2-(3-methoxyphenyl)ethyl]phenoxy]propyl]-4-methylpiperazine	565	
**SC124**	2-[5-[2-[2-(3-methoxyphenyl)ethyl]phenoxy]pentyl]-3,4-dihydro-1H-isoquinoline	>1000	
**SC125**	6,7-dimethoxy-2-[5-[2-[2-(3-methoxyphenyl)ethyl]phenoxy]pentyl]-3,4-dihydro-1H-isoquinoline	>1000	
**SC126**	2-[2-[2-[2-(3-methoxyphenyl)ethyl]phenoxy]ethyl]-3,4-dihydro-1H-isoquinoline	>1000	
**SC127**	4-(1H-benzimidazol-2-ylmethyl)-2-(2-phenylethyl)morpholine	>100	[90]

[2] All K_I values retrieved from ChEMBL were determined in vitro utilizing radio-ligand binding assays.

3. Theoretical evaluation of generated pharmacophore models

3.1. M1 – Structure-based LigandScout model

Table S3. Quantitative evaluation of the optimization process involved in the development of the final SB LS pharmacophore model M1. V1 to V9 represent the preliminary versions throughout the model development process. Optimization progress was based on model accuracy, yield of actives and enrichment factor. Actives, inactives and decoys represent the number of training compounds found by the intermediate / final model. TP, true positives. FP, false positives. TN, true negatives. DB, database. Sens., Sensitivity. Spec., Specificity. YoA, Yield of actives. EF, Enrichment factor.

Model	Actives (n = 68)	Inactives (n = 68)	Decoys (n = 3752)	TP	FP	TN	Sens.	Spec.	Accuracy	YoA	EF
V1	2	1	19	2	20	3800	0.03	0.99	0.98	0.09	5.20
V2	2	1	30	2	31	3789	0.03	0.99	0.98	0.06	3.47
V3	10	3	307	10	310	3510	0.15	0.92	0.91	0.03	1.79
V4	12	4	410	12	414	3406	0.18	0.89	0.88	0.03	1.61
V5	12	4	388	12	392	3428	0.18	0.90	0.88	0.03	1.70
V6	11	3	326	11	329	3491	0.16	0.91	0.90	0.03	1.85
V7	10	2	263	10	265	3555	0.15	0.93	0.92	0.04	2.08
V8	12	4	388	12	392	3428	0.18	0.90	0.88	0.03	1.70
V9	9	1	75	9	76	3744	0.13	0.98	0.97	0.11	6.05
**M1**	9	1	35	9	36	3784	0.13	0.99	0.98	0.20	11.44

3.2. M2 – Structure-based DiscoveryStudio model

Table S4. Quantitative evaluation of the optimization process involved in the development of the final SB DS pharmacophore model M2. V1 to V14 represent the preliminary versions throughout the model development process. Optimization progress was based on model accuracy, yield of actives and enrichment factor. Actives, inactives and decoys represent the number of training compounds found by the intermediate / final model. TP, true positives. FP, false positives. TN, true negatives. DB, database. Sens., Sensitivity. Spec., Specificity. YoA, Yield of actives. EF, Enrichment factor.

**Model**	**Actives (n = 68)**	**Inactives (n = 68)**	**Decoys (n = 3752)**	**TP**	**FP**	**TN**	**Sens.**	**Spec.**	**Accuracy**	**YoA**	**EF**
V1	16	4	357	16	361	3459	0.24	0.91	0.89	0.04	2.43
V2	12	1	246	12	247	3573	0.18	0.94	0.92	0.05	2.65
V3	17	0	216	17	216	3604	0.25	0.94	0.93	0.07	4.17
V4	17	0	128	17	128	3692	0.25	0.97	0.95	0.12	6.70
V5	17	0	93	17	93	3727	0.25	0.98	0.96	0.15	8.84
V6	17	0	128	17	128	3692	0.25	0.97	0.95	0.12	6.70
V7	14	0	72	14	72	3748	0.21	0.98	0.97	0.16	9.31
V8	17	0	65	17	65	3755	0.25	0.98	0.97	0.21	11.85
V9	17	0	59	17	59	3761	0.25	0.98	0.97	0.22	12.79
V10	17	0	55	17	55	3765	0.25	0.99	0.97	0.24	13.50
V11	17	0	55	17	55	3765	0.25	0.99	0.97	0.24	13.50
V12	16	0	48	16	48	3772	0.24	0.99	0.97	0.25	14.29
V13	16	0	42	16	42	3778	0.24	0.99	0.98	0.28	15.77
V14	16	0	38	16	38	3782	0.24	0.99	0.98	0.30	16.94
**M2**	16	0	37	16	37	3783	0.24	0.99	0.98	0.30	17.26

3.3. *M3 – Ligand-based LigandScout model*

Table S5. Quantitative evaluation of the optimization process involved in the development of the final ligand-based LS pharmacophore model M3. V1 to V14 represent the preliminary versions throughout the model development process. Optimization progress was based on model accuracy, yield of actives and enrichment factor. Actives, inactives and decoys represent the number of training compounds found by the intermediate / final model. TP, true positives. FP, false positives. TN, true negatives. DB, database. Sens., Sensitivity. Spec., Specificity. YoA, Yield of actives. EF, Enrichment factor.

**Model**	**Actives (n = 68)**	**Inactives (n = 68)**	**Decoys (n = 3752)**	**TP**	**FP**	**TN**	**Sens.**	**Spec.**	**Accuracy**	**YoA**	**EF**
V1	8	1	654	8	655	3165	0.12	0.83	0.82	0.01	0.69
V2	6	2	528	6	530	3290	0.09	0.86	0.85	0.01	0.64
V3	6	0	136	6	136	3684	0.09	0.96	0.95	0.04	2.42
V4	9	1	170	9	171	3649	0.13	0.96	0.94	0.05	2.86
V5	4	0	33	4	33	3787	0.06	0.99	0.98	0.11	6.18
V6	8	0	19	8	19	3801	0.12	1.00	0.98	0.30	16.94
V7	6	0	5	6	5	3815	0.09	1.00	0.98	0.55	31.19
V8	27	3	32	27	35	3785	0.40	0.99	0.98	0.44	24.90
V9	24	1	23	24	24	3796	0.35	0.99	0.98	0.50	28.59
V10	27	3	70	27	73	3747	0.40	0.98	0.97	0.27	15.44
V11	26	1	40	26	41	3779	0.38	0.99	0.98	0.39	22.19
V12	27	1	46	27	47	3773	0.40	0.99	0.98	0.36	20.86
V13	27	1	22	27	23	3797	0.40	0.99	0.98	0.54	30.88
V14	26	1	16	26	17	3803	0.38	1.00	0.98	0.60	34.57
**M3**	26	1	9	26	10	3810	0.38	1.00	0.99	0.72	41.29

3.4. *M4 – Ligand-based DiscoveryStudio model*

Table S6. Quantitative evaluation of the optimization process involved in the development of the final ligand-based DS pharmacophore model M4. V1 to V15 represent the preliminary versions throughout the model development process. Optimization progress was based on model accuracy, yield of actives and enrichment factor. Actives, inactives and decoys represent the number of training compounds found by the intermediate / final model. TP, true positives. FP, false positives. TN, true negatives. DB, database. Sens., Sensitivity. Spec., Specificity. YoA, Yield of actives. EF, Enrichment factor.

**Model**	**Actives (n = 68)**	**Inactives (n = 68)**	**Decoys (n = 3752)**	**TP**	**FP**	**TN**	**Sens.**	**Spec.**	**Accuracy**	**YoA**	**EF**
V1	34	1	466	34	467	3353	0.50	0.88	0.87	0.07	3.88
V2	34	1	466	34	467	3353	0.50	0.88	0.87	0.07	3.88
V3	32	1	424	32	425	3395	0.47	0.89	0.88	0.07	4.00
V4	32	0	268	32	268	3552	0.47	0.93	0.92	0.11	6.10
V5	31	0	185	31	185	3635	0.46	0.95	0.94	0.14	8.21
V6	30	0	169	30	169	3651	0.44	0.96	0.95	0.15	8.62
V7	29	0	137	29	137	3683	0.43	0.96	0.95	0.17	9.99
V8	30	0	120	30	120	3700	0.44	0.97	0.96	0.20	11.44
V9	30	0	110	30	110	3710	0.44	0.97	0.96	0.21	12.25
V10	30	0	87	30	87	3733	0.44	0.98	0.97	0.26	14.66
V11	30	0	55	30	55	3765	0.44	0.99	0.98	0.35	20.18
V12	30	0	56	30	56	3764	0.44	0.99	0.98	0.35	19.95
V13	30	0	36	30	36	3784	0.44	0.99	0.98	0.45	25.99
V14	30	0	32	30	32	3788	0.44	0.99	0.98	0.48	27.67
V15	30	0	29	30	29	3791	0.44	0.99	0.98	0.51	29.07
**M4**	30	0	28	30	28	3792	0.44	0.99	0.98	0.52	29.57

4. Selection of compounds after virtual screening – allocation to the different pharmacophore models

Table S7. Pharmacophore model origins of the selected virtual hits after Ro5 and PAINS filtering. Hits selected based on the different pharmacophore models M1, 2, 3 and 4 are marked with an X. Marked in yellow were compounds with NDF values ≥ 2, thus, originally considered for K_I determination, but were discarded after literature research. Novel D2R ligands were marked in green.

Compound ID	M1	M2	M3	M4	Compound ID	M1	M2	M3	M4
10		X	X	X	SC169		X		
SC128			X		SC170			X	
SC129			X		SC171		X		X
14			X	X	SC172		X		
SC130			X		SC173				X
SC131			X	X	SC174	X			
SC132			X	X	SC175		X		
11	X	X		X	SC176	X			
SC133				X	SC177			X	
SC134	X	X			SC178			X	X
SC135				X	SC179			X	
SC136			X	X	SC180			X	X
SC137		X			SC181		X		X
SC138		X		X	SC182			X	
SC139		X		X	SC183		X		X
SC140		X			SC184			X	X
SC141			X		SC185	X			
SC142		X			SC186			X	X
SC143				X	SC187			X	X
SC144				X	SC188	X			
SC145				X	16		X		X
SC146				X	12	X	X		X
SC147		X		X	SC189	X			X
SC148				X	SC190	X			X
SC149		X			17	X	X		
SC150				X	SC191		X		
SC151	X				SC192	X			
SC152	X				SC193			X	
SC153	X				18		X		
SC154		X	X		SC194			X	X
SC155		X			SC195		X		
SC156		X		X	SC196			X	X
SC157				X	SC197				X
SC158			X		SC198		X		X
SC159				X	SC199		X		X
SC160		X			SC200	X			
15		X	X		SC201		X		
SC161		X		X	SC202	X			
SC162	X			X	SC203	X			
SC163	X				SC204				X
SC164	X				19			X	X
SC165				X	SC205		X		X
SC166		X			SC206	X			
SC167		X			SC207		X		
SC168		X		X	SC208	X	X		

5. Similarity assessment – comparison of compounds from virtual hit groups

Table S8. Comparison of the most similar compounds selected from each of the virtual hit groups. Similarity analysis is based on TS calculated from radial fingerprints. Only the 2D structures of the most similar hits from each of the respective groups are shown. TS, Tanimoto score.

Pharmacophore model	TS	2D structures	
M1 (compounds **SC185** and **SC192**)	0.25	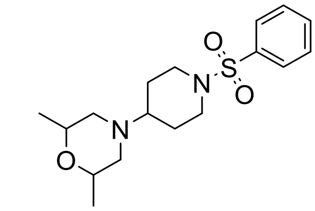	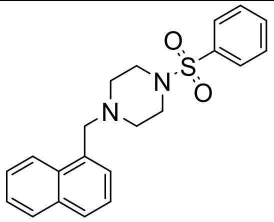
M2 (compounds **SC169** and **SC175**)	0.27	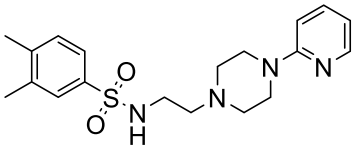	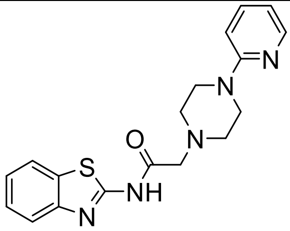
M3 (compounds **SC131** and **SC136**)	0.19	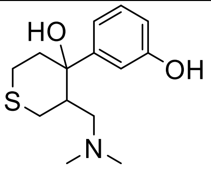	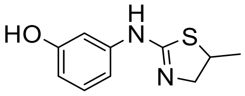
M4 (compounds **SC146** and **SC148**)	0.26	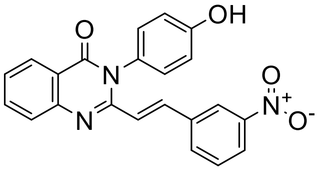	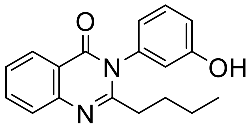
M2 + M4 (compounds **16** and **12**)	0.26	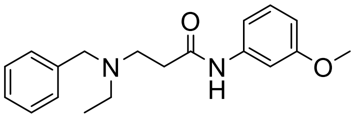	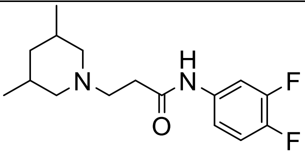
M4 + M1 / M3 (compounds **SC180** and **SC196**)	0.33	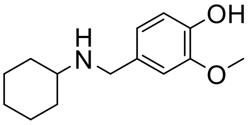	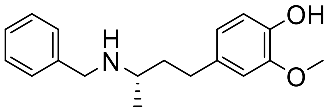
M2 + M1 / M3 (compounds **SC154** and **15**)	0.33	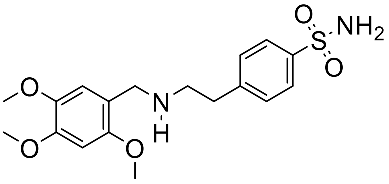	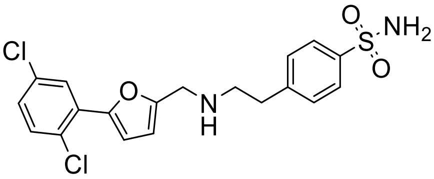
M2 + M4 + M1 / M3 (compounds **11** and **12**)	0.12	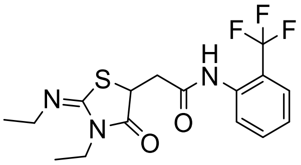	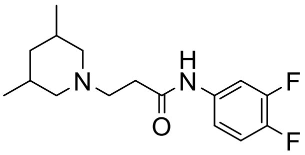

6. Hit selection after virtual screening – overview of 2D structures and NDF values of all compounds

Table S9. Overview of all virtual hits selected for further in vitro investigations resulting from the in silico screening of SPECS and Maybridge databank with the generated pharmacophore models. 2D structures and NDF ± standard deviation (SD) are shown for each compound (n = 4). NDF, normalized decreased fluorescence.

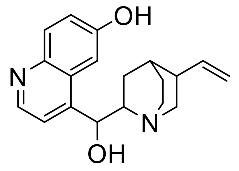

10

NDF = 1.13 ± 0.37

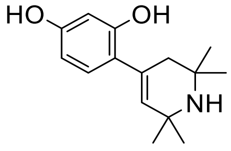

SC128

NDF = 1.05 ± 0.45

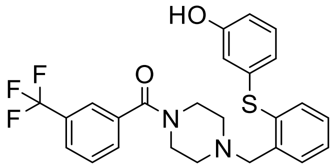

SC129

NDF = 0.90 ± 0.25

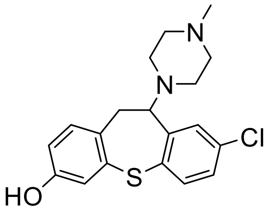

14

NDF = 40.41 ± 1.39

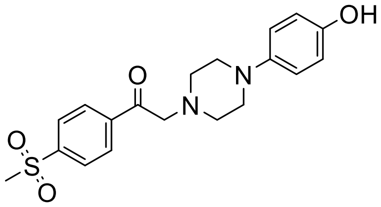

SC130

NDF = 1.01 ± 0.38

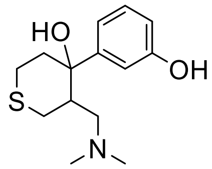

SC131

NDF = 0.90 ± 0.30

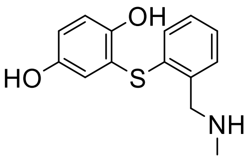

SC132

NDF = 0.91 ± 0.36

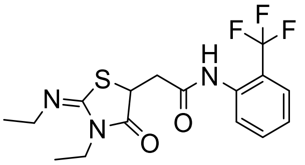

(11)

NDF = 0.84 ± 0.23

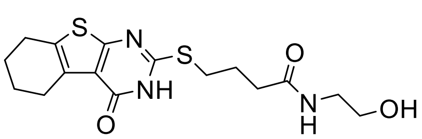

SC133

NDF = 0.95 ± 0.43

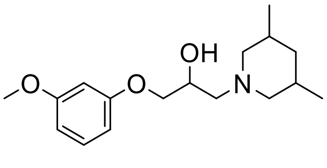

SC134

NDF = 1.02 ± 0.39

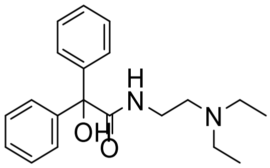

SC135

NDF = 1.01 ± 0.50

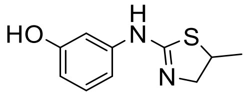

SC136

NDF = 0.95 ± 0.43

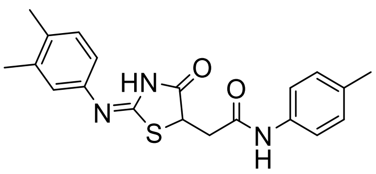

SC137

NDF = 0.94 ± 0.32

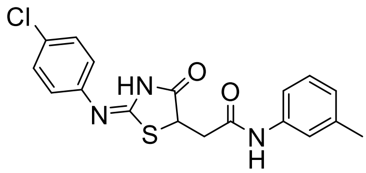

SC138

NDF = 0.96 ± 0.38

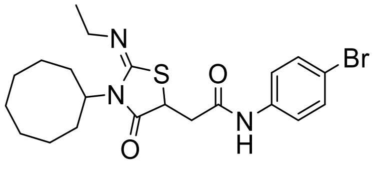

SC139

NDF = 1.00 ± 0.35

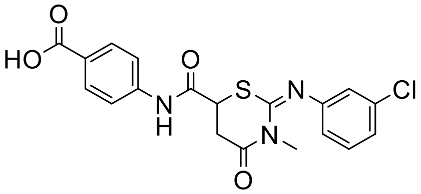

SC140

NDF = 0.82 ± 0.25

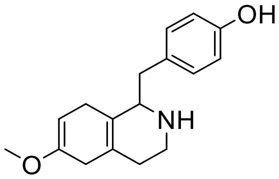

SC141

NDF = 1.14 ± 0.56

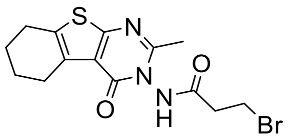

SC142

NDF = 0.99 ± 0.35

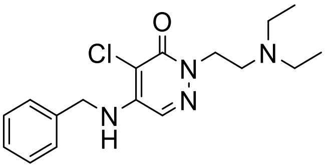

SC143

NDF = 0.99 ± 0.39

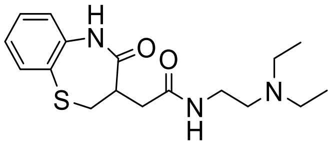

SC144

NDF = 1.02 ± 0.36

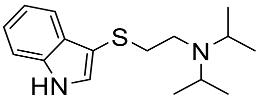

SC145

NDF = 0.98 ± 0.32

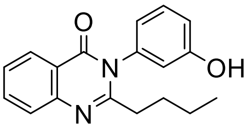

SC146

NDF = 0.93 ± 0.29

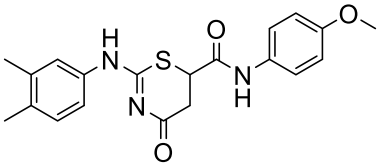

SC147

NDF = 0.94 ± 0.36

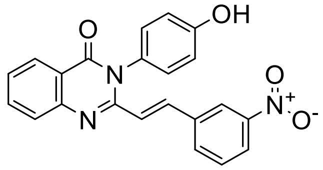

SC148

NDF = 0.86 ± 0.24

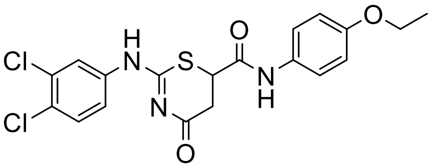

SC149

NDF = 2.04 ± 1.34

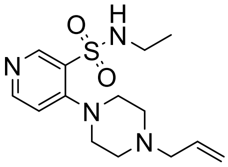

SC150

NDF = 1.10 ± 0.46

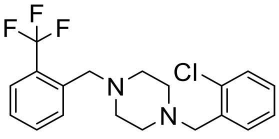

SC151

NDF = 1.06 ± 0.41

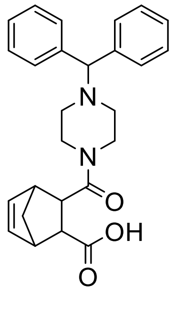

SC152

NDF = 1.02 ± 0.35

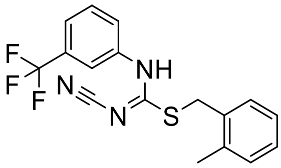

SC153

NDF = 1.10 ± 0.44

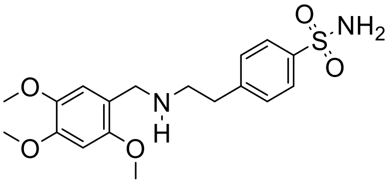

SC154

NDF = 0.94 ± 0.32

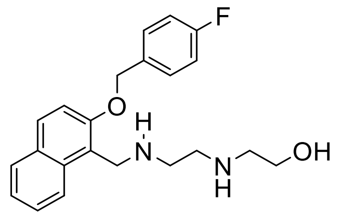

SC155
NDF = 1.68 ± 0.90

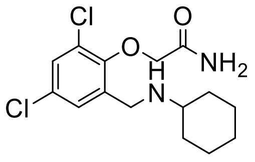

SC156
NDF = 0.95 ± 0.32

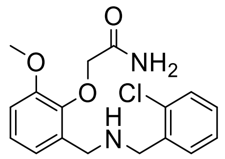

SC157
NDF = 1.01 ± 0.44

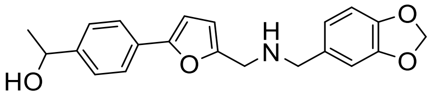

SC158
NDF = 0.93 ± 0.42

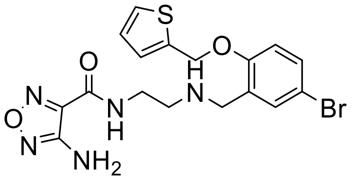

SC159
NDF = 1.14 ± 0.57

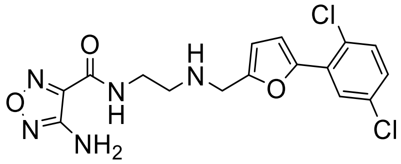

SC160
NDF = 2.41 ± 2.01

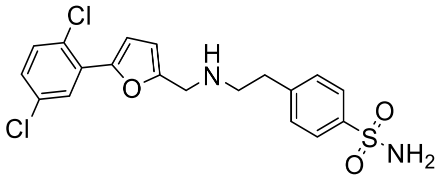

15
NDF = 3.99 ± 2.58

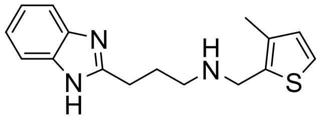

SC161
NDF = -

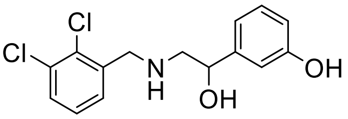

SC162
NDF = 0.90 ± 0.38

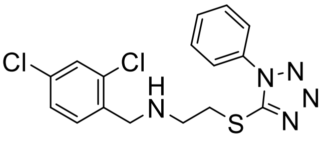

SC163
NDF = 0.97 ± 0.36

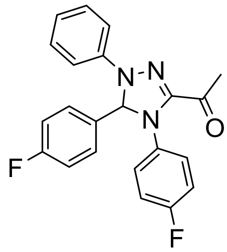

SC164
NDF = 0.90 ± 0.25

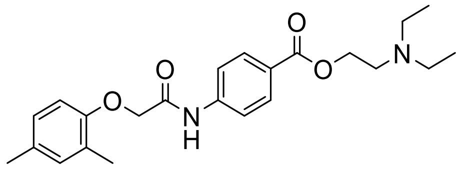

SC165
NDF = 0.95 ± 0.35

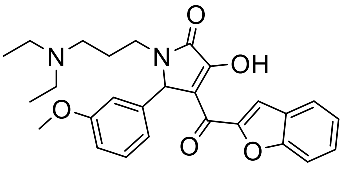

SC166
NDF = -

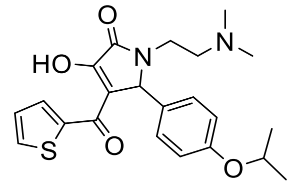

SC167
NDF = 0.94 ± 0.46

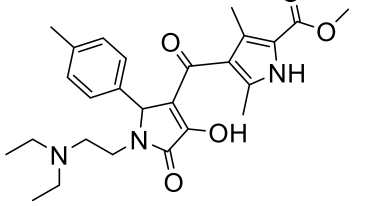

SC168
NDF = 0.95 ± 0.42

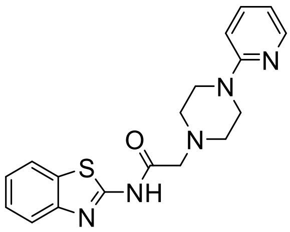

SC169
NDF = 0.90 ± 0.39

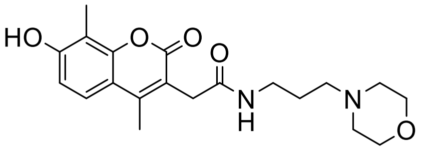

SC170
NDF = 0.99 ± 0.43

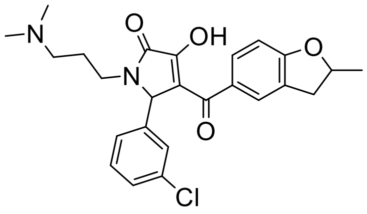

SC171
NDF = -

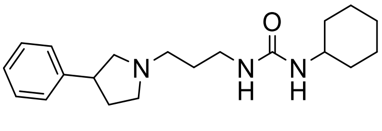

SC172
NDF = 1.35 ± 0.68

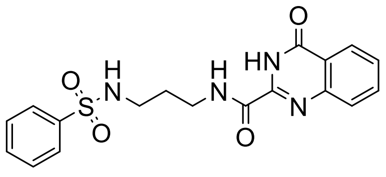

SC173
NDF = 0.90 ± 0.35

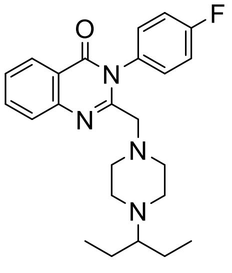

SC174
NDF = 0.90 ± 0.36

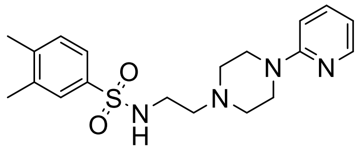

SC175
NDF = 2.98 ± 1.77

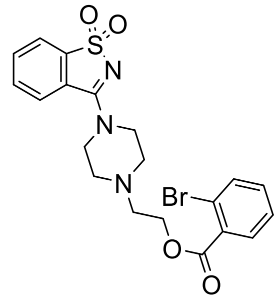

SC176
NDF = 0.96 ± 0.37

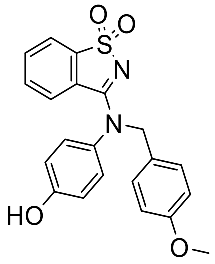

SC177
NDF = 1.03 ± 0.42

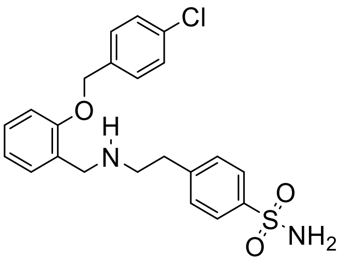

SC178
NDF = 1.07 ± 0.46

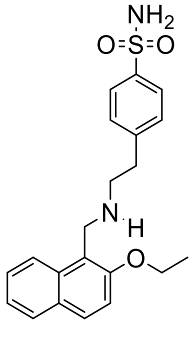

SC179
NDF = 0.99 ± 0.40

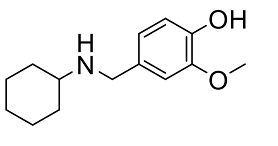

SC180
NDF = 0.93 ± 0.38

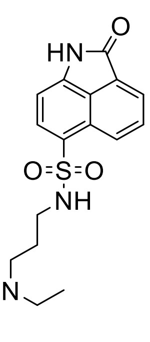

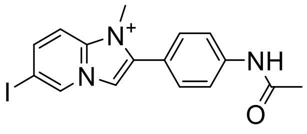

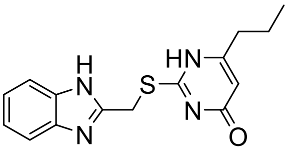

SC181
NDF = 0.94 ± 0.41

SC182
NDF = 1.41 ± 1.07

SC183
NDF = 1.52 ± 1.15

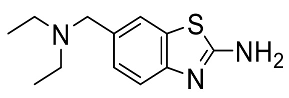

SC184
NDF = 1.38 ± 0.91

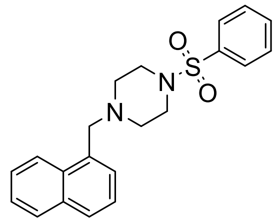

SC185
NDF = 1.06 ± 0.44

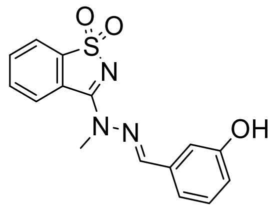

SC186
NDF = 1.06 ± 0.42

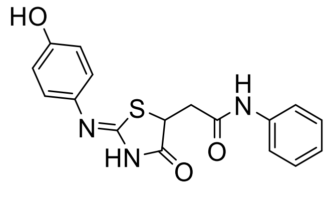

SC187
NDF = 0.95 ± 0.38

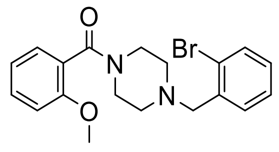

SC188
NDF = 0.97 ± 0.39

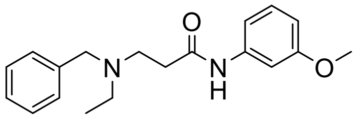

16
NDF = 15.74 ± 18.15

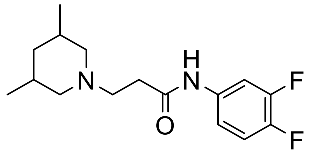

12
NDF = 1.05 ± 0.50

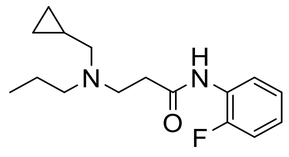

SC189
NDF = 1.02 ± 0.38

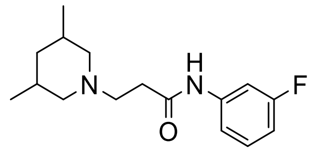

SC190
NDF = 1.26 ± 0.70

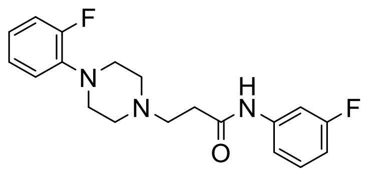

17
NDF = 8.18 ± 3.62

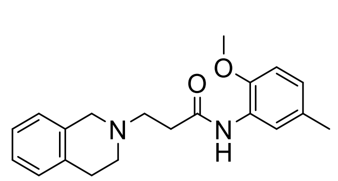

SC191
NDF = 1.83 ± 0.83

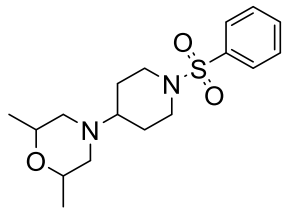

SC192
NDF = 0.99 ± 0.42

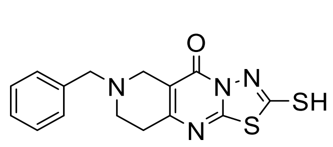

SC193
NDF = 1.02 ± 0.47

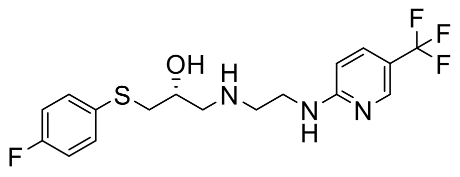

18
NDF = 10.85 ± 4.93

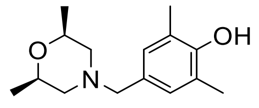

SC194
NDF = 1.00 ± 0.40

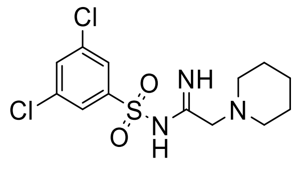

SC195
NDF = 0.99 ± 0.39

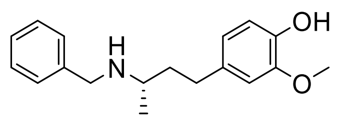

SC196
NDF = 1.10 ± 0.52

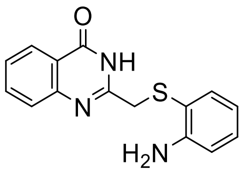

SC197
NDF = 1.13 ± 0.34

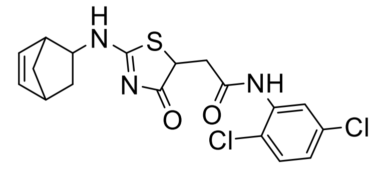

SC198
NDF = 2.44 ± 1.39

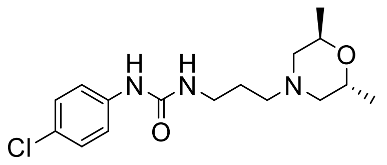

SC199
NDF = 1.66 ± 1.60

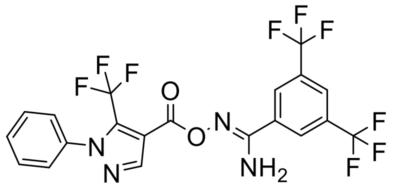

SC200
NDF = 1.11 ± 0.47

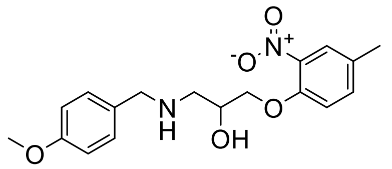

SC201
NDF = 2.00 ± 0.85

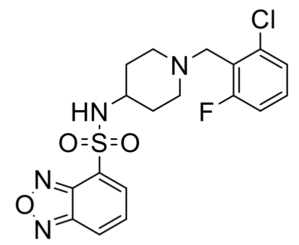

SC202
NDF = 0.93 ± 0.40

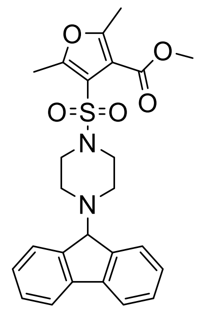

SC203
NDF = 1.11 ± 0.51

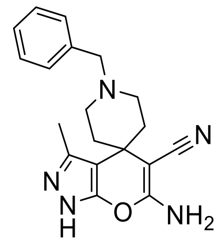

SC204
NDF = 1.41 ± 0.71

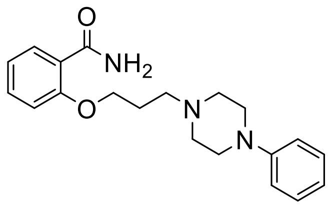

19
NDF = 22.08 ± 6.62

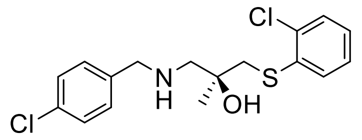

SC205
NDF = 1.11 ± 0.53

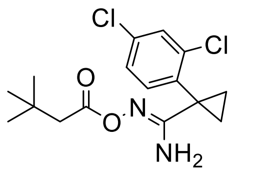

SC206
NDF = 1.08 ± 0.56

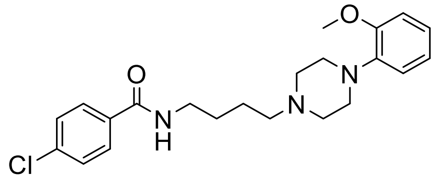

SC207
NDF = 22.89 ± 8.41

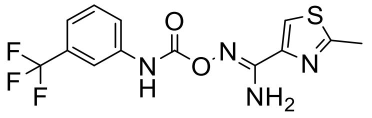

SC208
NDF = 1.04 ± 0.39

7. Compound selection after in vitro screening – literature analysis

Table S10. Ligand selection after in vitro screening of virtual hits. The structure as well as the normalized decrease of fluorescence ± standard deviation (SD) in comparison to the control is shown. All compounds were screened as biological duplicates (n = 4) at 10 µM. Compounds are sorted according to decreasing NDF. NDF, normalized decreased fluorescence.

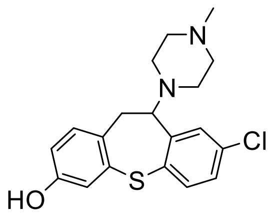

14

NDF = 40.40 ± 1.39

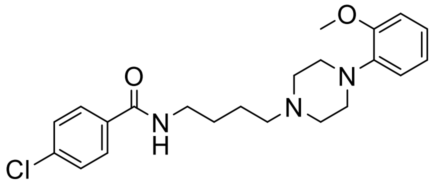

SC207

NDF = 22.89 ± 8.41

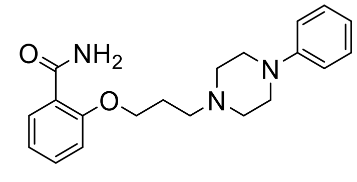

19

NDF = 22.08 ± 6.62

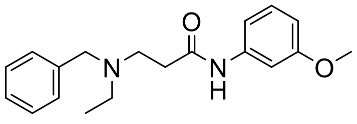

16

NDF = 15.74 ± 18.15

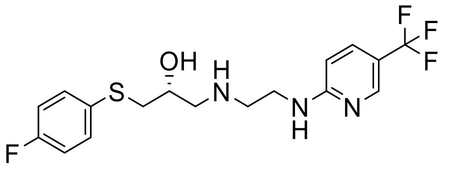

18

NDF = 10.85 ± 4.93

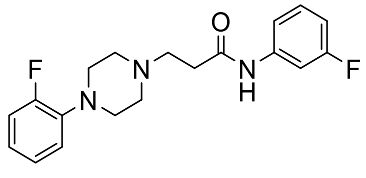

17

NDF = 8.18 ± 3.62

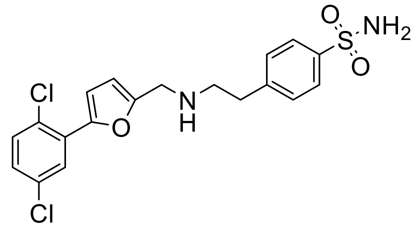

15

NDF = 3.99 ± 2.58

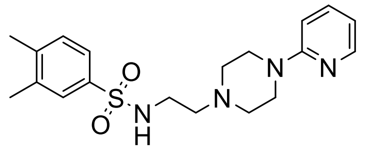

SC175

NDF = 2.98 ± 1.77

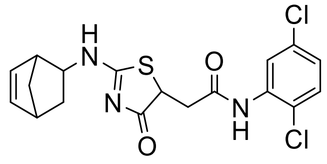

SC198

NDF = 2.43 ± 1.39

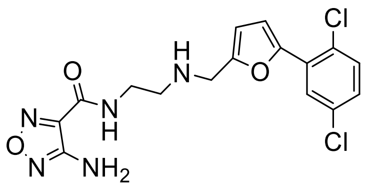

SC160

NDF = 2.41 ± 2.01

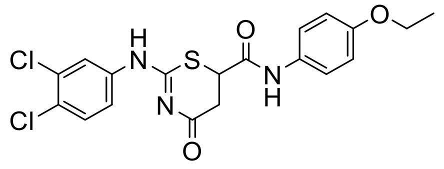

SC149

NDF = 2.04 ± 1.34

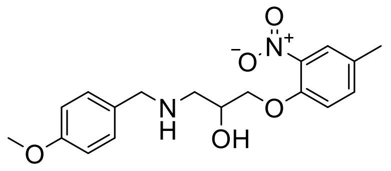

SC201

NDF = 1.99 ± 0.85

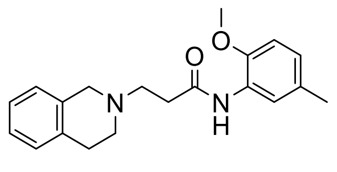

SC191

NDF = 1.83 ± 0.83

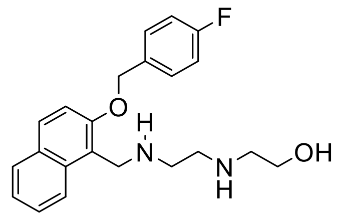

SC155

NDF = 1.68 ± 0.90

8. Similarity assessment – investigation of identified D$_2$R ligands

8.1. Comparison to data set of actives

Table S9. Assessment of scaffold diversity comparing identified D$_2$R ligands with the active training compounds (1 to 9 and SC1 to SC59) the pharmacophore models have been trained with. Comparison of 2D structures was based on the calculation of radial fingerprints, subsequently applying a TS matrix. Only the most similar compounds from the training set for each ligand is shown. TS, Tanimoto score.

Compound ID	TS	2D structures	
**14** (to **SC19**)	0.18	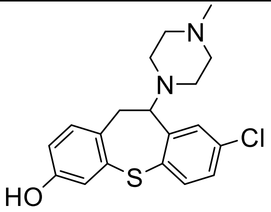	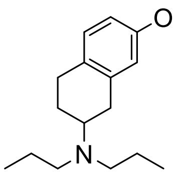
**15** (to **SC22**)	0.09	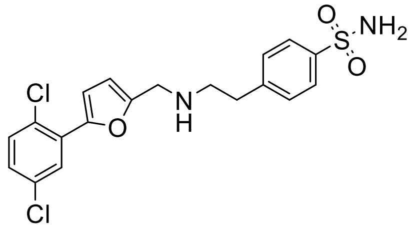	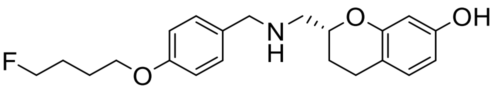
**16** (to **17**)<br>**17** (to **16**)	0.28	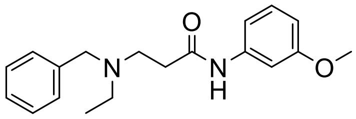	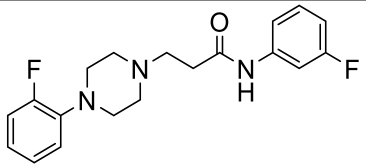
**18** (to **15**)	0.09	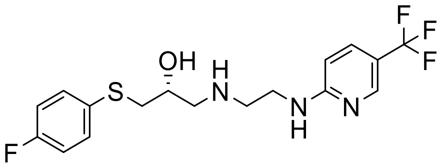	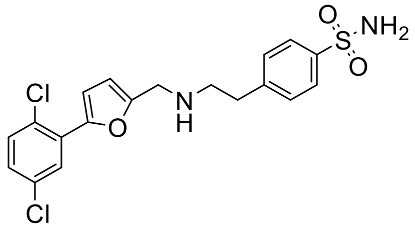
**19** (to **SC30**)	0.26	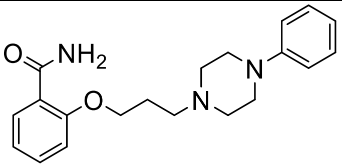	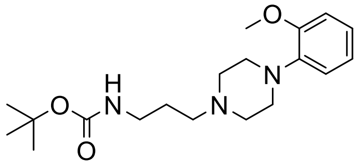

8.2. *Comparison to ChEMBL*

Table S10. Assessment of scaffold and compound novelty of the identified D2R ligands. Ligands were compared to known D2R ligands published in ChEMBL. For comparison, only active compounds with a binding affinity (K_I) of up to 500 nM were considered. Similarity in between different compounds was investigated using the Tanimoto coefficient (radial fingerprints based on compounds 2D structures). The most similar ChEMBL entries based on the TS regarding each novel ligand are shown. TS, Tanimoto score.

Compound ID	TS	2D structures	
**14** (to **SC209**)	0.58	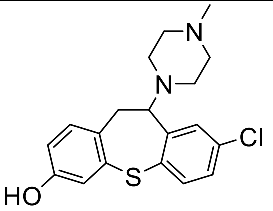	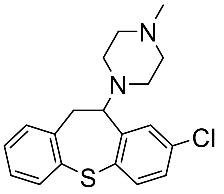
**15** (to **SC210**)	0.21	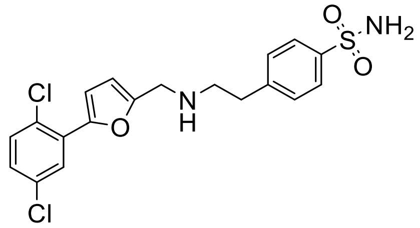	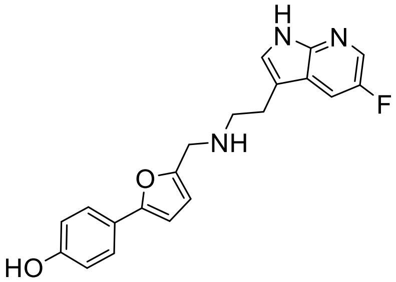
**16** (to **SC211**)	0.45	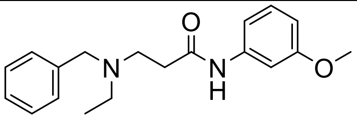	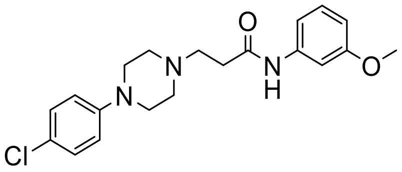
**17** (to **SC211**)	0.42	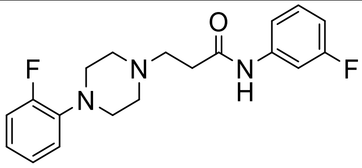	
**18** (to **SC212**)	0.19	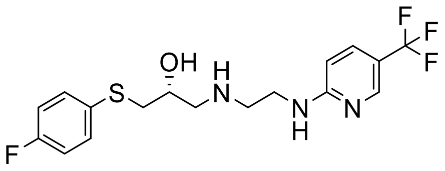	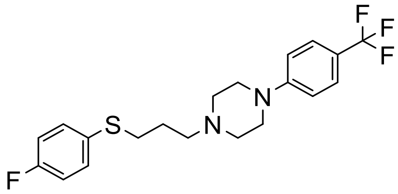
**19** (to **SC213**)	0.51	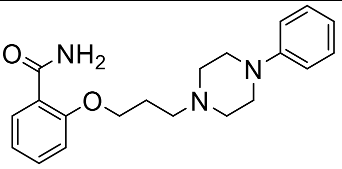	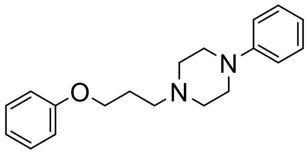

9. Pharmacophore models

PDB ID 6vms [22] also represents the cryo-EM structure of D2R bound to bromocriptine displaying the same binding mode. However, it was discarded before pharmacophore generation due to the lower resolution of the structure (2.8 Å vs 3.8 Å).

9.1. Display of the original structure-based pharmacophore models M1 and M2

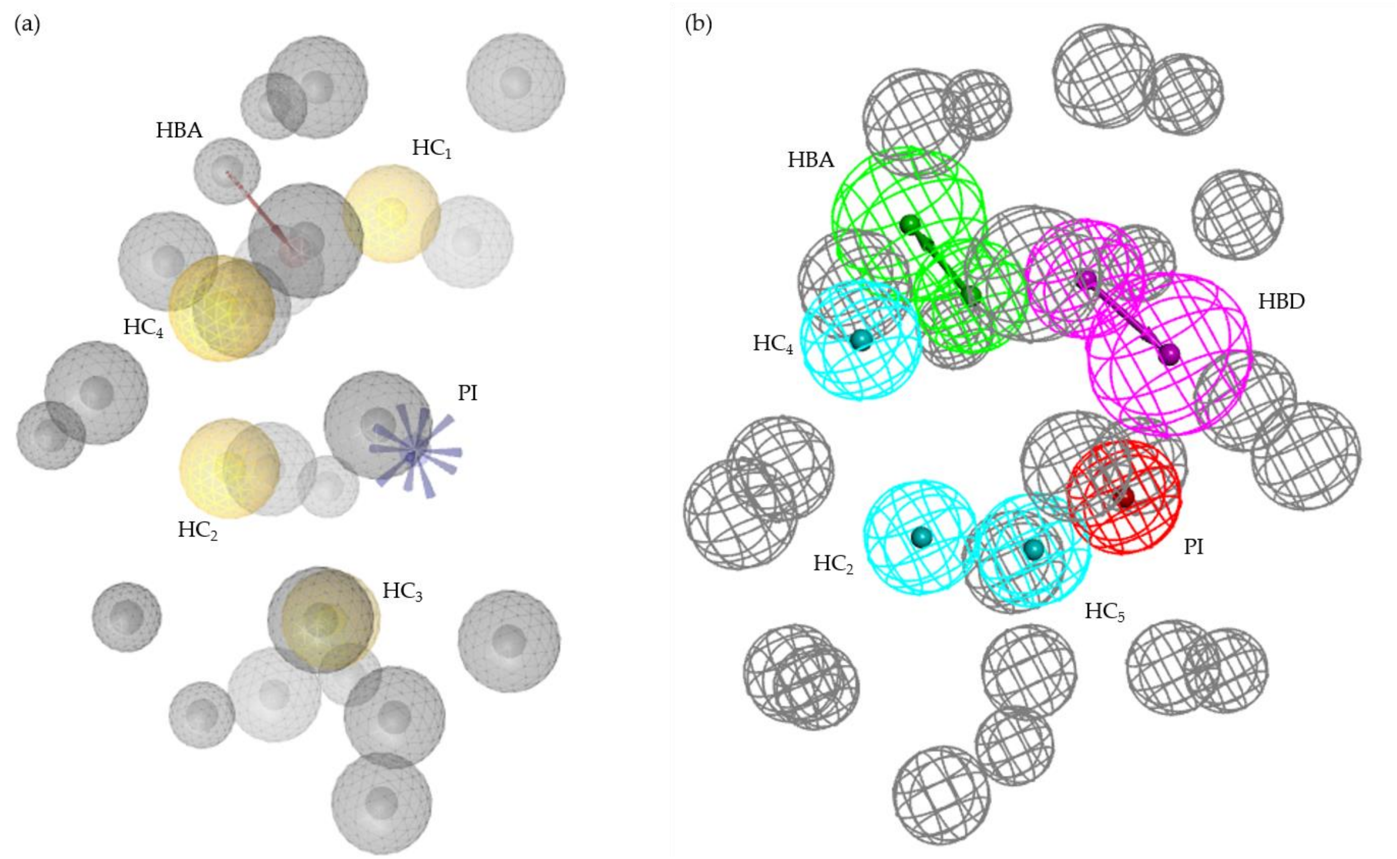

Figure S1. Display of the original SB pharmacophore models generated in (**a**) LS and (**b**) DS. Pharmacophores are shown after automatic model generation. Equivalent annotations of the features in between the different models indicated equivalent coordinates. Different feature annotations (highlighted by different indices) reflect different coordinates of the features, respectively. XVOLs (grey). Hydrophobic contacts (HC; yellow and cyan spheres). Hydrogen bond acceptor (HBA; red arrows, green spheres). Hydrogen bond donor (HBD; purple sphere). Positively ionizable interaction (PI; blue star-like, red sphere).

9.2. Selected compounds from the data set of actives – generation of ligand-based pharmacophore models M3 and M4

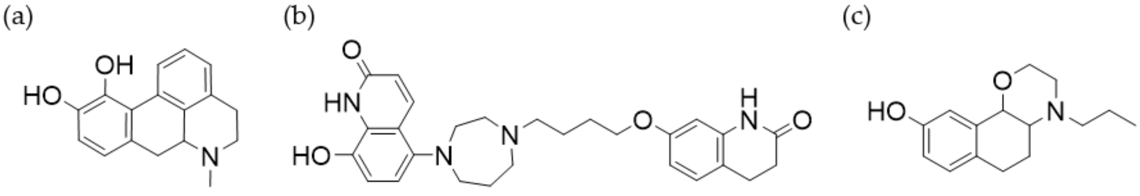

1 K_I = 1.8 nM **SC52** K_I = 1.6 nM **SC59** K_I = 0.45 nM

Figure S2. 2D structures of active data set compounds used for the generation of LB pharmacophore models. K_I values were extracted from the respective ChEMBL entries. (**a**) 2D structure of **1**. (**b**) 2D structure of **SC52**. (**c**) 2D structure of **SC59**.

9.3. *Display of the original ligand-based pharmacophore models M3 and M4*

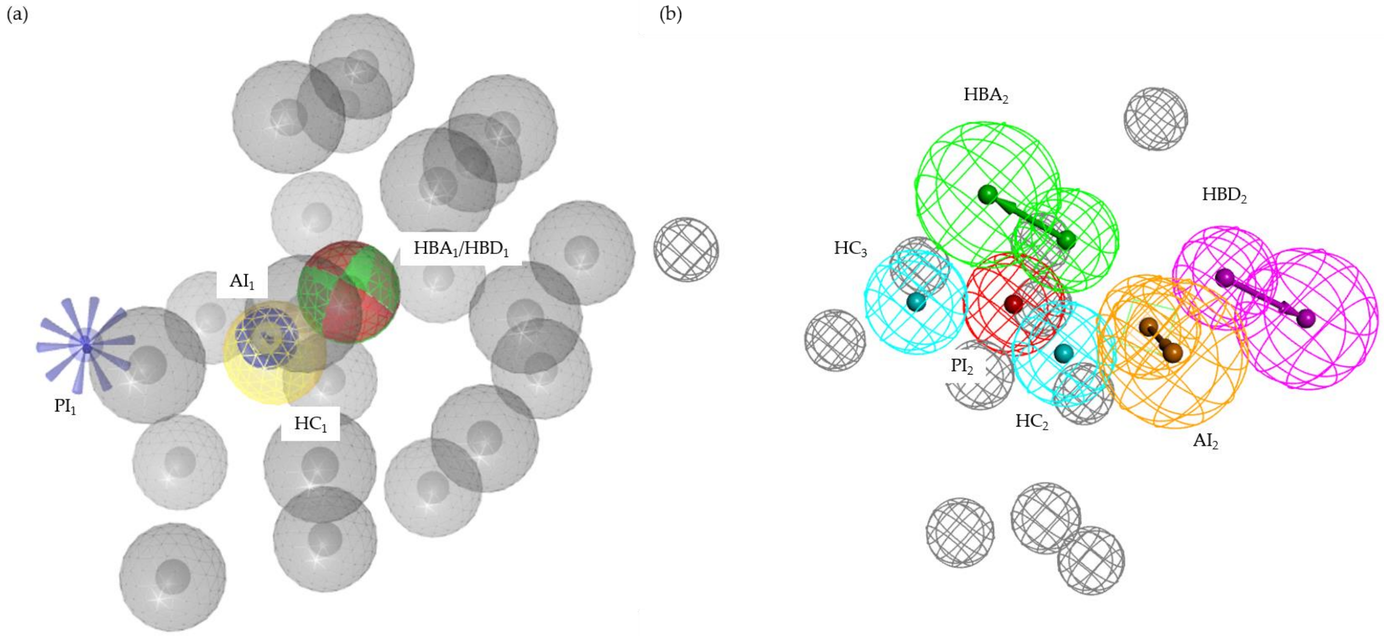

Figure S3. Display of the original LB pharmacophore models generated in (**a**) LS and (**b**) DS. Pharmacophores are shown after automatic model generation. XVOLs (grey). Hydrophobic contacts (HC; yellow and cyan spheres). Hydrogen bond acceptor (HBA; red and green spheres). Hydrogen bond donor (HBD; green and purple sphere). Positively ionizable interaction (PI; blue star-like, red sphere). Aromatic interaction (AI; blue circle, orange sphere).

9.4. *Assessing comparability of M3 and M4 based on feature location superimposed with compound SC59*

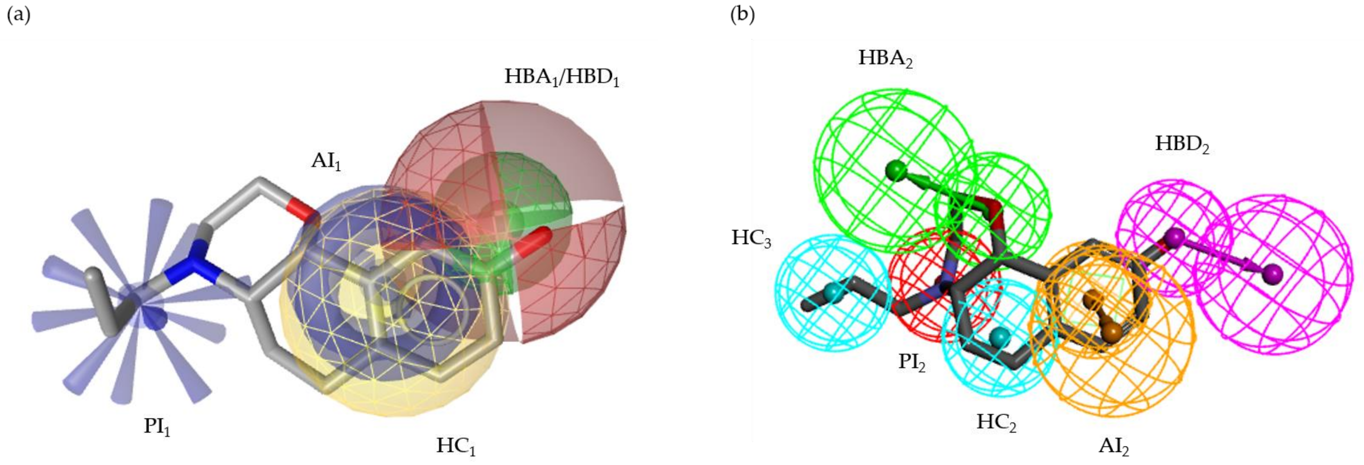

Figure S4. Alignment of **SC59** with the optimized LB pharmacophore models (**a**) M3 and (**b**) M4. Assessment of feature comparability based on the superpositioning of the pharmacophore models and compound 3D structure.

10. Tanimoto score matrices – similarity assessments in the different virtual hit groups

The selected virtual hits from each group, either single or consensus hits, were assessed considering their scaffold diversity. Therefore, radial fingerprints (ECFP4 [93, 94]) were generated from each compound based on its 2D structure. Subsequently, the fingerprints were compared applying the Tanimoto score [100]. The highest Tanimoto scores representing the most similar compounds in each of the groups are shown in Table S8. Additionally, the complete Tanimoto matrices for all groups are shown in Figure S5.

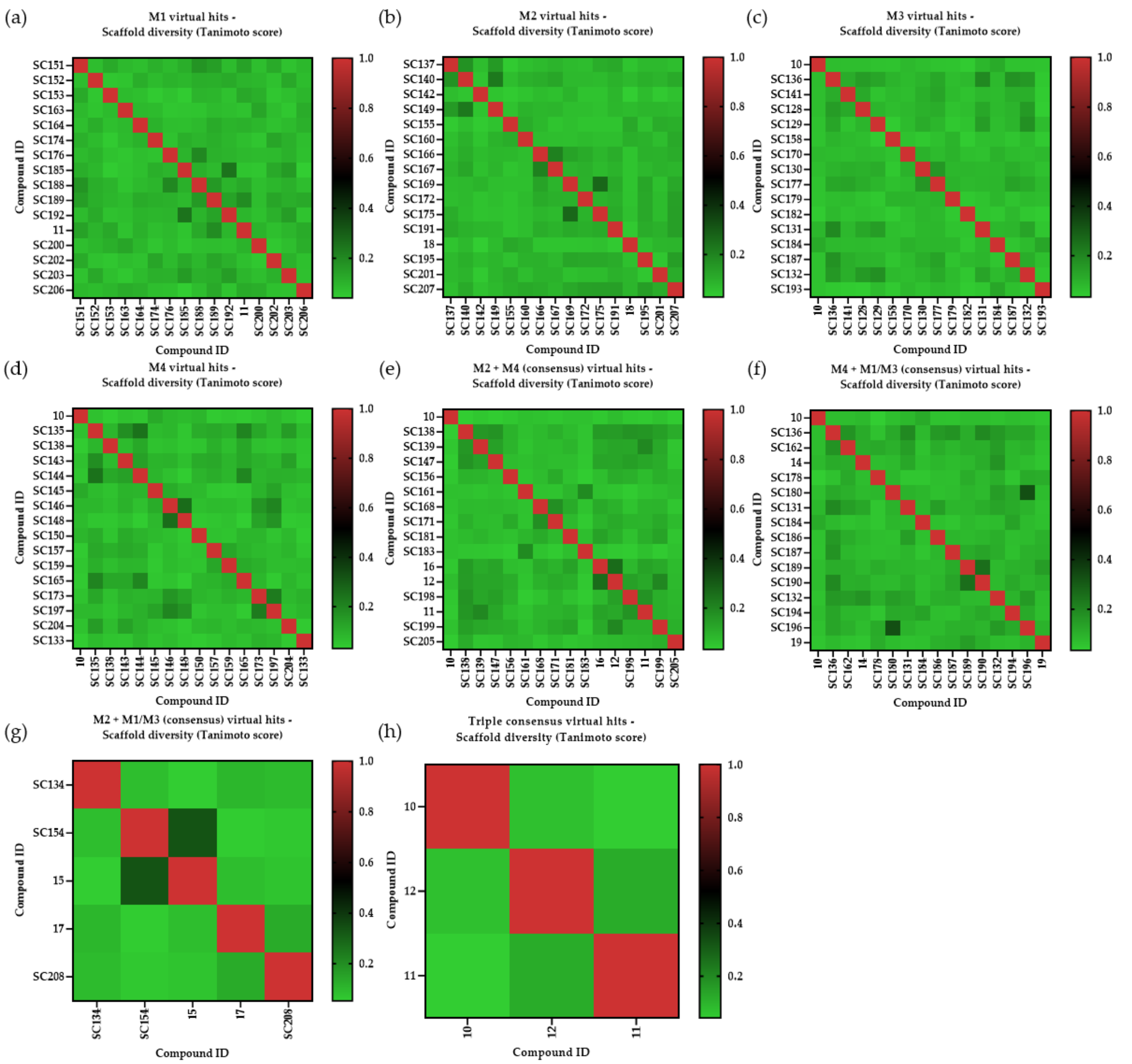

Figure S5. TS matrices comparing the selected virtual hits allocated to the single hit or consensus hit groups. (**a-d**) Comparison of the 16 selected ligands from each model, respectively. (**e**) Comparison of the 16 selected ligands from the double consensus hits including M2 and M4. (**f**) Comparison of the 16 selected ligands from the double consensus hits of the M4 + M1/M3 group. (**g**) Comparison of the 5 selected ligands from the double consensus hits of the M2 + M1/M3 group. (**h**) Comparison of the 3 selected ligands from the triple consensus hits. The comparison is based on ECFP4 radial fingerprints. The TS is ranging from 0 (green) to 1 (red) indicating increasing similarity in between the molecules. The matrices were calculated in Canvas 3.8 and illustrated in GraphPad Prism 8. TS, Tanimoto score.

11. In vitro analysis of selected virtual hits

11.1. Summary of in vitro activity (based on NDF values) of control compounds and selected virtual hits

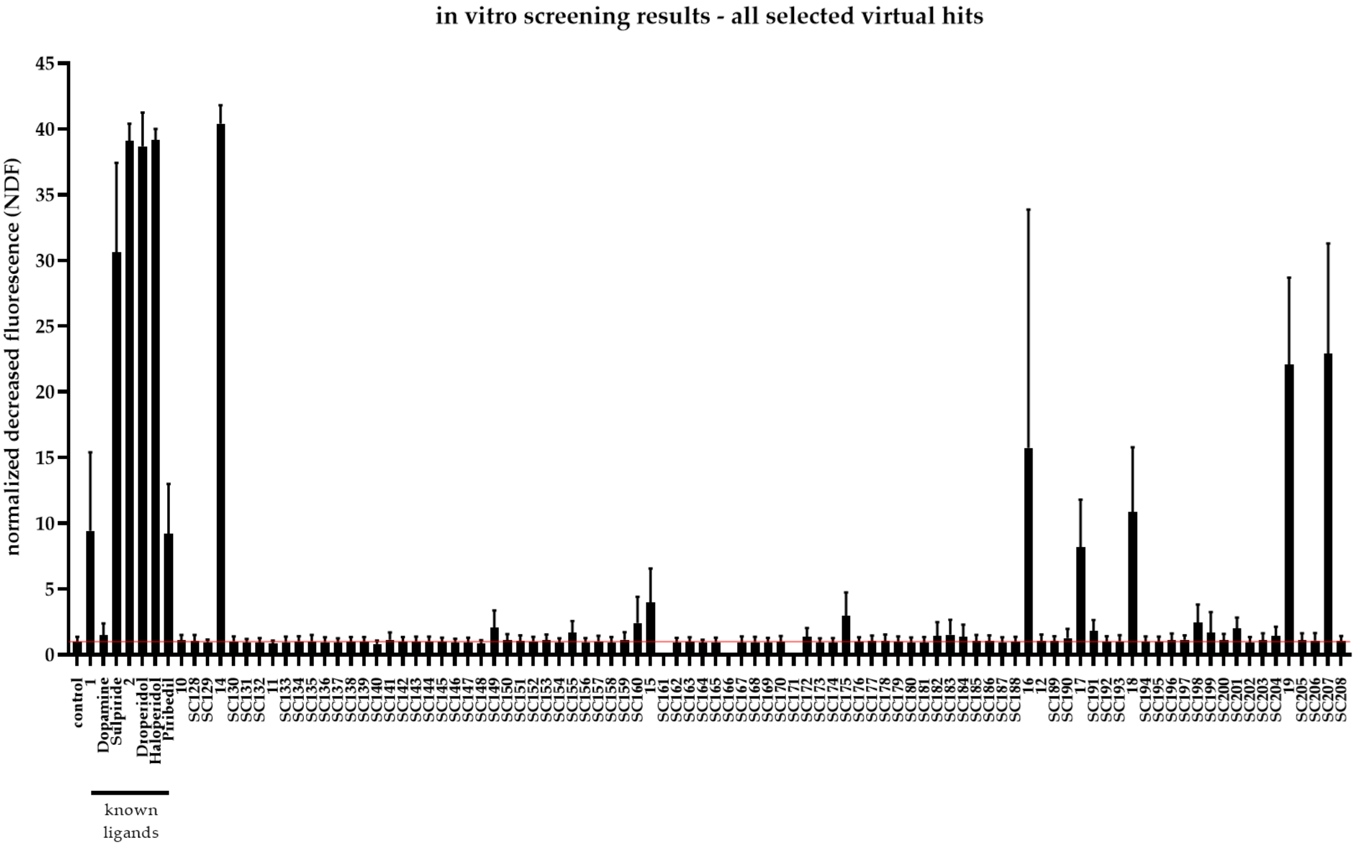

Figure S6. Screening of known D2R ligands (apomorphine, dopamine, sulpiride, bromocriptine, droperidol, haloperidol and piribedil) and all selected 90 virtual hits predicted by the in silico pharmacophore models as active ligands. Compound binding is shown as NDF in comparison to the control. Activities were measured in biological duplicates (two technical replicates each). Error bars show standard deviations (SD). The red line indicates a NDF of 1, thus, an inactive ligand at 10 μM screening concentration. NDF, normalized decreased fluorescence.

11.2. *Summary of K_I values determined in vitro – overview of identified D2R ligands*

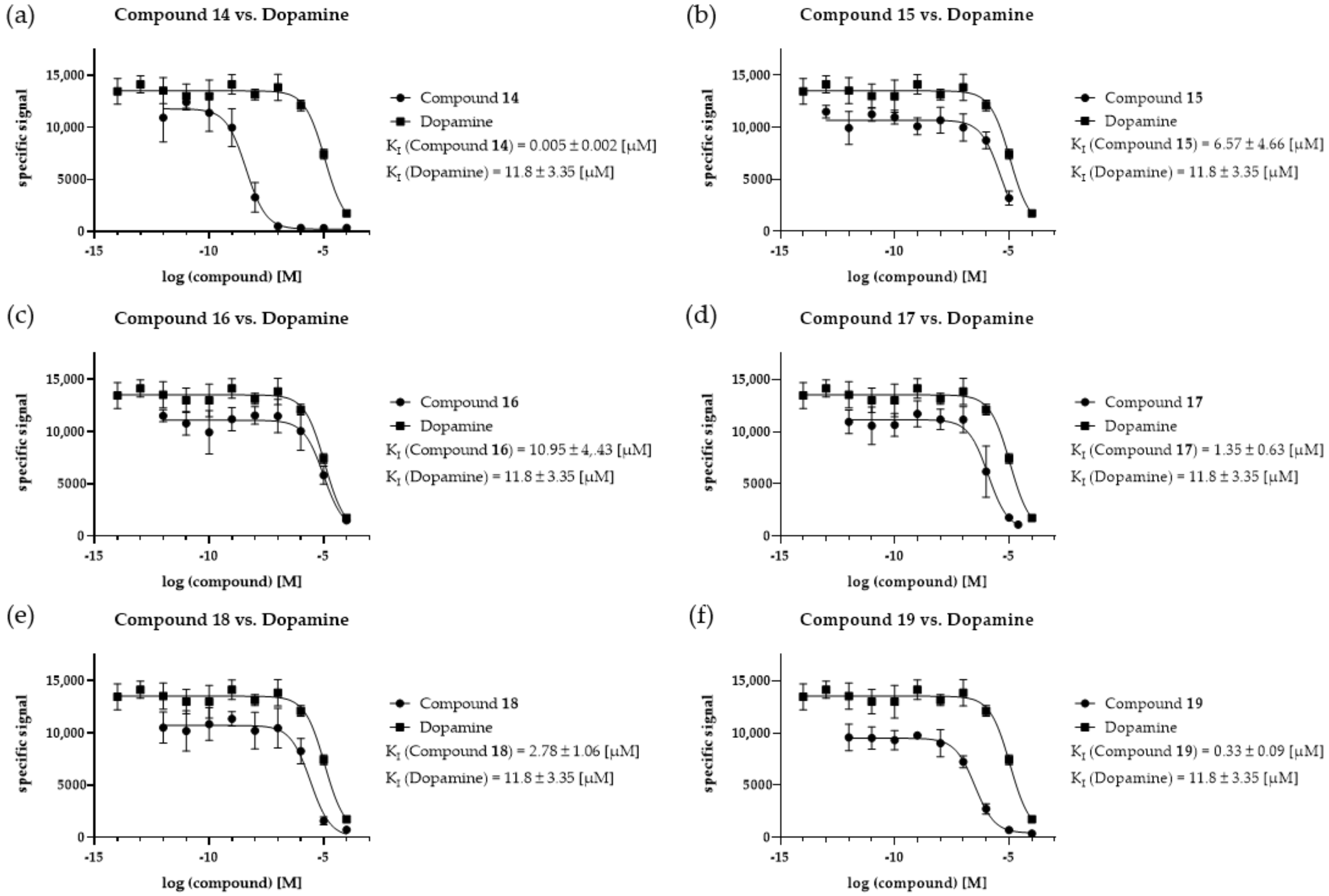

Figure S7. Comparison of K_I values of dopamine (endogenous ligand) and (**a**)-(**e**) all of the identified D2R ligands. Dopamine shows micromolar activity. The novel ligands exert either activities similar to dopamine (e.g. compound **16**) or exert a higher binding affinity towards the D2R. High binding affinities are defined by calculated fold-differences > 1 as shown in Table 4. K_I values were determined measuring biological duplicates of each compound. Each biological duplicate was measured in technical triplicates. The specific signal at each concentration includes ± standard deviations (SD).

12. Assessing scaffold diversity – novel ligands vs. active data set

The novel D2R ligands were assessed considering their scaffold diversity in comparison to the original training compounds used for the generation of the pharmacophore models. Therefore, radial fingerprints (ECFP4) were generated for each compound based on its 2D structure. Subsequently, fingerprints were compared applying the Tanimoto score. The highest Tanimoto score of each novel D2R ligand in comparison to the training set is shown in Table S11. Each compound is shown with the most similar compound from the training set according to their Tanimoto score Compounds **16** and **17** score 0.28. This score is due to the aromatic cycles at both ends of the structures, the amide functionality and the same length of the linker region in between the amide group and the tertiary amine. However, compound **16** possesses a simple tertiary amine while compound **17** is characterized by a pyrazine motif. The highest Tanimoto score comparing a novel D2R ligand and a training compound is shown in between compound **19** and **SC30** scoring 0.26. The similarity score is due to a piperazine functionality of both compounds adjacent to an aromatic cycle. However, functional groups (amide and carbamate) as well as the substituents differ from each other. Thus, the identified D2R ligands can be considered diverse from each other as well as from the original set of training compounds.

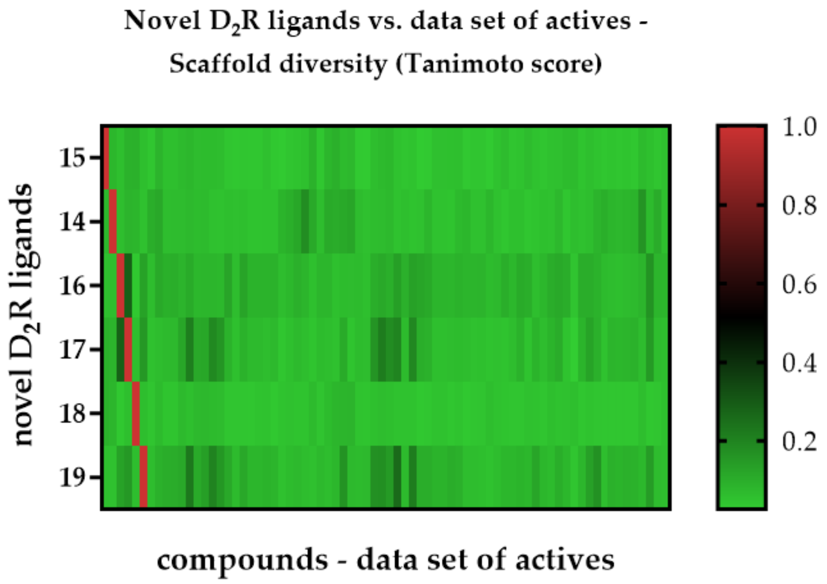

Figure S8. TS matrices comparing the identified D2R ligands with the active data set used during pharmacophore model generation. All novel ligands are indicated on the y-axis. Training compounds (left to right) are not annotated separately. The comparison is based on radial fingerprints (ECFP4). The TS is ranging from 0 (green) to 1 (red) indicating increasing similarity in between the molecules. The matrices were calculated in Canvas 3.8 and illustrated in GraphPad Prism 8. TS, Tanimoto score.

13. Identified D2R ligands – pharmacophore model alignments

The alignments of compound **15** with M3 and M2 are shown in Figure S9a and b. Additionally, the 2D structure of compound **15** including the various interactions defined by the pharmacophore models is shown in Figure S9c. All HB features originating from the two models are located around the sulfonamide group of the ligand. The overlapping AI / HC_1 features from the LS model are placed in the adjacent aromatic ring. The remaining HC features HC_2, HC_3 and HC_4 originate from the DS model and are located at the furane ring and at both chloro-substituents. This part of the molecule is not covered by the LS model. Again, both models are in agreement placing the PI at the central secondary amine.

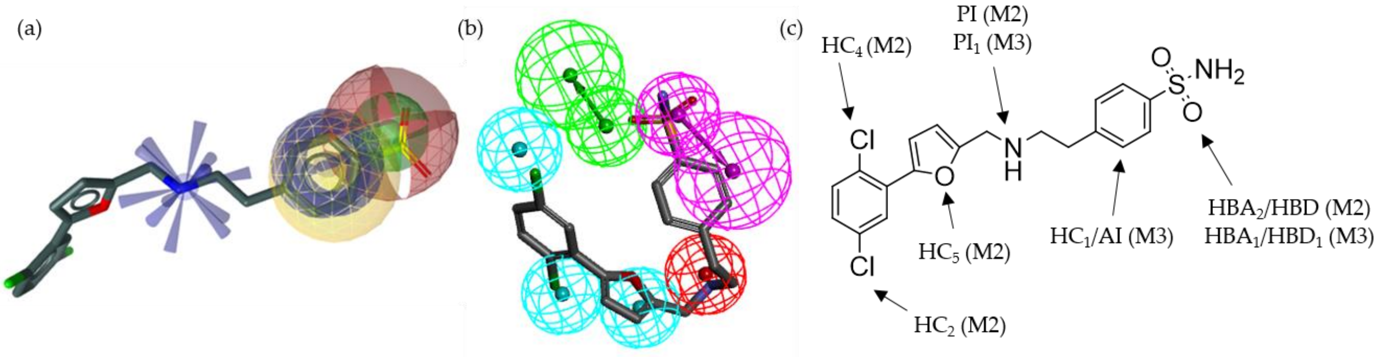

Figure S9. Display of the novel D2R ligand, compound **15**, aligned with M2 and M3 (**a**) 3D structure of compound **15** including M3 features. (**b**) 3D structure of compound **15** including M2 features. (**c**) 2D structure of compound **15** highlighting structural features recognized by the pharmacophore models.

The alignments of compound **16** with the M4 and M2 are shown in Figure S10a and b. Additionally, the 2D structure of compound **16** including the various interactions defined by the pharmacophore models is shown in Figure S10c. While the models originate from different modelling approaches, they all place the features on the same key functionalities of the novel ligand. HC_1 and HC_2 are present in both models and located at the amine bound aromatic ring and the ethyl-substituent of the tertiary amine respectively. HC_3 is only present in the structure-based approach located at the methoxy group of compound **16**. Both HB features are located around the amide functional group. The AI feature only

present in the ligand-based model is centered at the methoxy-substituted aromatic ring. Again, both models agree in placing the PI at the tertiary amine.

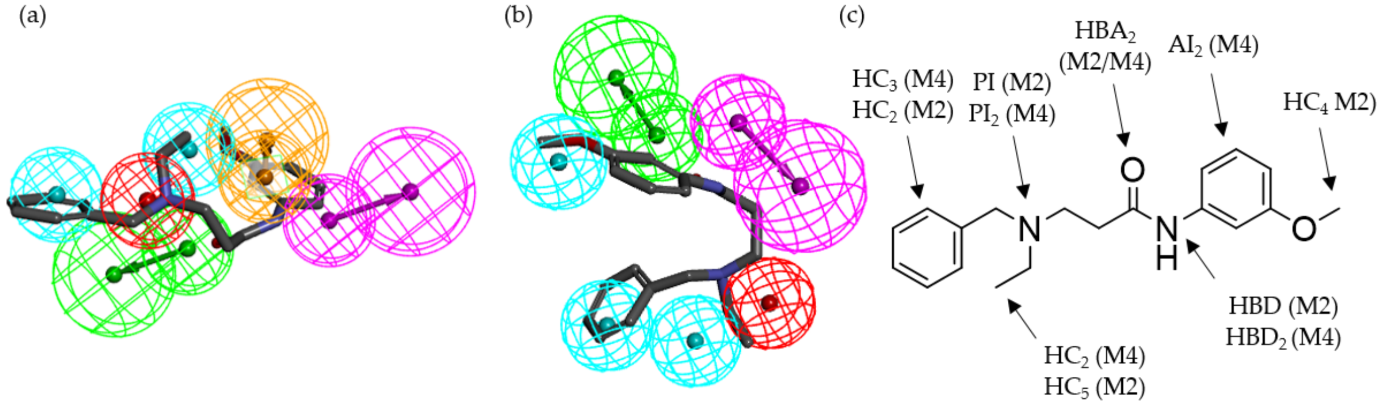

Figure S10. Display of the novel D_2R ligand, compound **16**, aligned with M2 and M4. (**a**) 3D structure of compound **16** including M4 features. (**b**) 3D structure of compound **16** including M2 features. (**c**) 2D structure of compound **16** highlighting structural features recognized by the pharmacophore models.

The alignments of compound **17** with M1 and M2 are shown in Figure S11a and b. The 2D structure of compound **17** including the various interactions de-fined by the pharmacophore models is shown in Figure S11c. Both models feature three different HCs located in similar regions comparing the models. HC_1 and HC_2 are located around the fluoro-substituted aromatic ring adjacent to the piperazine motif. HC_3 is located at the other end of the molecule at an analogous fluoro-substituted aromatic ring adjacent to the amide functional group. HB_1 and HB_2 originate from M2 and are both centered around the amide functional group of the ligand. In contrast, HB_3 is present only in M1 located at the fluoro substituent neighboring the amide functional group. Both models are in agreement placing the PI at the more central tertiary amine group of the piperazine motif.

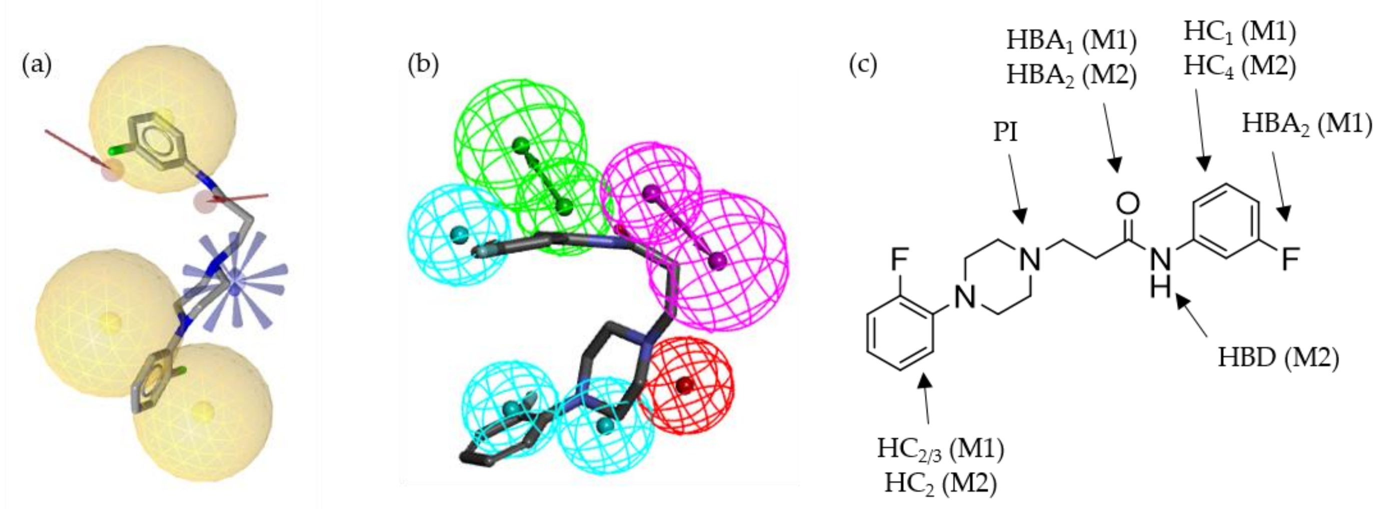

Figure S11. Display of the novel D_2R ligand, compound **17**, aligned M1 and M2. (**a**) 3D structure of compound **17** including M1 features. (**b**) 3D structure of compound **17** including M2 features. (**c**) 2D structure of compound **17** highlighting structural features recognized by the pharmacophore models.

Compound **18** was only identified by M2. Thus, all features originate from the same model. The alignment is shown in Figure S12a while the 2D structure of the ligand is displayed in Figure S12b. The HC features 1, 2 and 3 are located at the trifluoric substituent, the pyridine ring and the fluoro-substituted aromatic ring respectively. HB_1 and HB_2 are placed at the thio-ether functional group and the hydroxyl substituent. The PI feature is shared in between the two different secondary amines. Thus, the PI might be present at either one of them during ligand-receptor interaction.

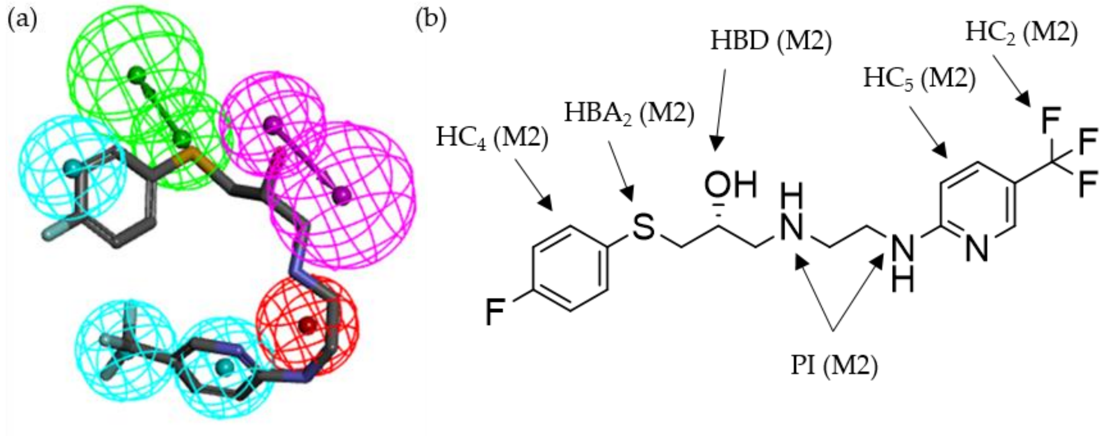

Figure S12. Display of the novel D2R ligand, compound **18**, aligned with M2. (**a**) 3D structure of compound **18** including M2 features. (**b**) 2D structure of compound **18** highlighting structural features recognized by the pharmacophore model.

The alignments of compound **19** with M3 and M4 are shown in Figure S13a and b. Additionally, the 2D structure of compound **19** including the various interactions defined by the pharmacophore models is shown in Figure S13c. Both models place AI_1, AI_2, HC_1 and HC_2 at the aromatic ring located in between amide and ether functional groups. HC_3 is present only in M4 placing it at the other end of the molecule at the benzyl ring. Again, the PI is located at the central nitrogen of the piperazine group of the novel ligand.

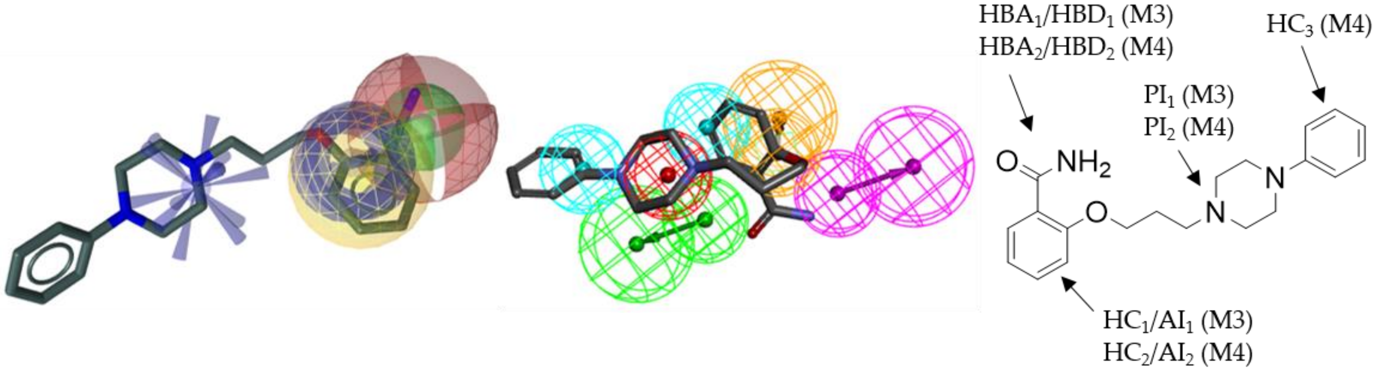

Figure S13. Display of the novel D2R ligand, compound **19**, aligned with M3 and M4 identifying the compound during the screening process. (**a**) 3D structure of compound **19** including M3 pharmacophore features. (**b**) 3D structure of compound **19** including the M4 pharmacophore features. (**c**) 2D structure of compound **19** highlighting structural features recognized by the pharmacophore models.
